# Barriers and Enablers to Integrating Patient-Generated Health Data in Shared Decision-Making From Health Care Professional and Patient Perspectives: Scoping Review

**DOI:** 10.2196/85197

**Published:** 2026-08-10

**Authors:** Pavithren V S Pakianathan, Devender Kumar, Jayathissa Prabath, Rada Hussein, Josef Niebauer, Albrecht Schmidt, Jan Smeddinck

**Affiliations:** 1 Human-Centered Ubiquitous Media Group, Faculty of Mathematics, Informatics and Statistics, LMU Munich, Munich, Germany; 2 Ludwig Boltzmann Institute for Digital Health and Prevention, 22 Lindhofstrasse, Salzburg, 5020, Austria, 43 5725582701; 3 The Maersk Mc-Kinney Moller Institute, The Faculty of Engineering, University of Southern Denmark, Odense, Denmark; 4 University of Applied Sciences Technikum Wien, Vienna, Austria; 5 University Institute of Sports Medicine, Prevention and Rehabilitation, Paracelsus Medical University, Salzburg, Salzburg, Austria

**Keywords:** patient-generated health data, PGHD, scoping review, data integration, shared decision-making, digital health, digital biomarkers, personal informatics, technologies, digital health interventions, mHealth

## Abstract

**Background:**

Advances in sensor technologies and increased adoption of wearables and smartphones by individuals have led to an abundance of patient-generated health data (PGHD). This data, when used effectively, could help to further augment the process of shared decision-making (SDM) to enable patient-centered care. However, the possible integration and usage of PGHD introduces complexities and challenges, which warrant considering both health care professional (HCP) and patient perspectives.

**Objective:**

Summarize the relevant works from the past 10 years that reflect the perspectives of both HCPs and patients as key stakeholders on potential barriers and enablers to the integration of PGHD for SDM. We analyzed both perspectives to surface key challenges and opportunities with integrating PGHD throughout patient journeys, as well as clinical workflows.

**Methods:**

Electronic searches were done using 3 databases: PubMed, ACM Digital Library, and IEEE Xplore for papers published between March 2013 and March 2023. Enablers and barriers mentioned by the stakeholders in included papers were extracted and analyzed using thematic analysis. An existing six-stage workflow model for integrating PGHD was used as a reference for deductive coding. Subsequently, considering barriers and enablers faced by both the HCPs and patients uncovered various tensions and alignments of perspectives, which could be addressed in future work and can inform concepts, designs, and development in PGHD for SDM.

**Results:**

A total of 53 publications were included in the scoping review. Six main overarching themes for barriers and enablers were identified: (1) patient-provider relationship, (2) patient characteristics, (3) organizational factors, (4) medical ethics and law, (5) data-driven workflow, and (6) design and technology. The 6-stage workflow was further expanded based on the new findings to include 4 additional stages, which include contextual considerations outside of traditional clinical environments. In addition to partially corroborating previously established barriers in the 6-stage workflow model, several new barriers and enablers were identified throughout all stages. This model helps to further align the needs of HCPs and patients beyond the clinical setting and could benefit system designers who plan to integrate PGHD for SDM.

**Conclusions:**

This scoping review demonstrates that there are several factors to consider for effectively integrating PGHD into health-related SDM. Notably, such factors extend outside the boundaries of traditional clinical settings. Although there is agreement between HCPs and patients on certain factors, there are also tensions to be addressed. Our augmented 10-stage workflow model offers system designers an overview of the challenges and enablers to consider while designing for PGHD integration in clinical workflows and patient journeys to improve SDM.

## Introduction

### Overview

The challenges of aging populations and noncommunicable diseases across society have led to increased demands and stress on health care systems. Digital health technologies are seen as an enabler to solving such challenges. A review of telemedicine for chronic diseases revealed that wearable devices can help improve the management of chronic conditions by allowing for continuous monitoring of vital signs and reducing patient loads from hospitals [1], thereby improving the delivery of health care services. However, digital health technologies and consumer health care technologies are still facing hurdles in entering the integrated clinical market due to several factors such as infrastructure, technical training, and legal and ethics-related barriers [2]. Digital health technologies pave the path toward more personalized health care, and a key enabler to this end is the use of patient-generated health data (PGHD).

The rise of the Quantified Self movement led by people who track many kinds of data about themselves [3,4], followed by the mass market adoption of self-tracking technologies, coupled with advancements in mobile sensing technologies, has led to an increase in patients bringing their health data to clinical consultations via patient-initiated or clinician-initiated tracking [5,6]. PGHD refers to data created, recorded, or gathered by patients (or by family members or other caregivers) to help address one’s health concerns [7]. Notably, PGHD excludes data from electronic health records (EHRs), records entered by health care professionals (HCPs), and patient-reported outcomes. The integration of PGHD has been known to bring about benefits such as (1) *better contextualization of symptoms* [8], (2) *better clinical decision-making* [9], and (3) *personalized and participatory care* [10,11].

Although PGHD has the potential to play a key role in the digitalization of health care, several challenges [12] lie in the path toward the adoption of PGHD as a foundation for enabling novel and additional insights and applications in clinical practice. Unlike traditional clinical data, which is typically produced through the actions of HCPs in tightly controlled clinical conditions, PGHD are usually gathered throughout situated daily living under strongly varying and individual circumstances. This poses challenges for the interpretation and reliability of PGHD with varying quality [13], but also opens opportunities in the form of “objective data windows” into daily living that would otherwise not be available. Akin to medical and health information being available through internet search [14], PGHD integration in patient journeys and clinical workflows is likely to impact medical examination and shared decision-making (SDM)—the collaborative process between an HCP and a patient to make health-related decisions [15,16] serving as a cornerstone of person-centered care.

We posit that the integration of PGHD in the health care context has resulted in a complex sociotechnical system where PGHD could traverse throughout the health data lifecycle [17] and be interacted with by multiple actors—humans and machines—resulting in the emerging field of human-data interaction [18]. Given the vital role PGHD plays in improving SDM [19] and empowering patients in proactively managing their health, it is essential to understand the needs and perspectives of patients and HCPs on how it can be integrated into care pathways.

Over the past years, prior works have investigated PGHD from diverse perspectives—how individuals track data [20], how HCPs (can better) integrate data in clinical workflows [12], and how patients and HCPs collaborate with data [21]. However, there is a gap in holistically evaluating the needs and values of the key stakeholders—patients and HCPs—along patient journeys and HCPs’ clinical workflows. Using an ecological lens to surface system-level interactions and reduce misunderstandings between stakeholders [6,21], our scoping review aims to map how patients and HCPs perceive the integration of PGHD within SDM processes across patient journeys and clinical workflows.

The main contributions of this scoping review paper are as follows: (1) a 10-stage workflow for aligning HCP and patient perspectives on the common barriers and enablers while integrating PGHD across patient journeys and clinical workflows, (2) mitigation strategies to overcome barriers across the patient journeys and clinical workflows, and (3) aligning areas of tension between HCP and patients across the data-enabled clinical workflows and patient data journeys and corresponding alignment strategies.

**Figure 1.**
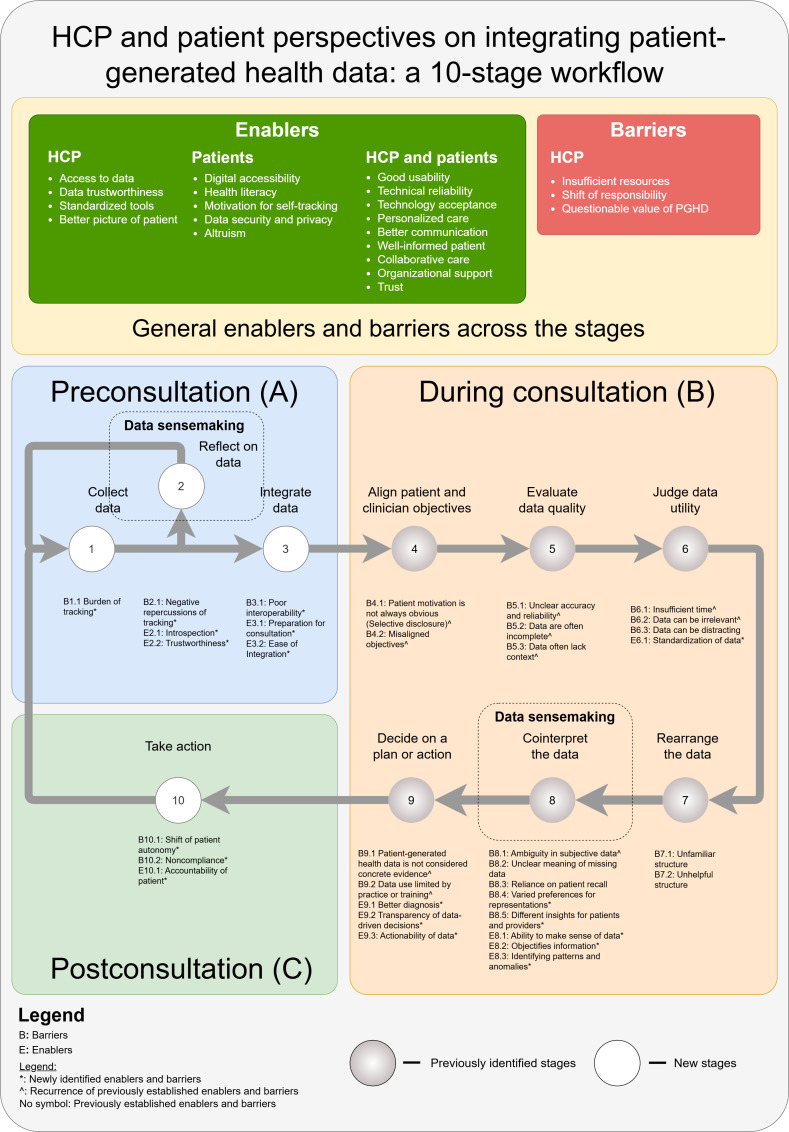
The adapted 10-stage workflow for integrating PGHD. HCP: health care professional; PGHD: patient-generated health data.

We use the findings of this scoping review to formulate a model—cf. 10-stage workflow model in Figure 1—which designers and health care technologists can use to analyze or pave data-enabled patient journeys and clinical workflows. By creating better data-enabled patient journeys, relationships between patients and HCPs can be improved, whereby patients could easily share information with HCPs and deliberate their preferences while participating in SDM [16].

### Background

#### Overview

We drew on prior work on PGHD and patient-HCP collaboration with PGHD. This sets the context on how technology has influenced the relationship between patients and HCPs, resulting in both alignments and tensions on integrating PGHD across patient data journeys and clinical workflows.

#### PGHD

The Quantified Self movement, powered by the rise of technological advancements and increased accessibility to wearables, has led to increased patients bringing opportunistically captured PGHD to consultations [5,22]. Q*uantifiers*—those who are part of the Quantified Self—are motivated to understand more about themselves through numbers and modify their lives accordingly [23]. They engage in such practices by using tracking technologies to self-collect personal data, including physiological, behavioral, or environmental information [24,25]. On the other hand, patients might engage in clinician-initiated tracking, which is purposefully capturing PGHD while receiving treatment or instructed by HCPs for long-term chronic care.

PGHD has also been enabled by remote monitoring technologies, which allow HCPs to issue technologies such as wearables and smartphone apps to patients who can take part in long-term treatment (eg, for postoperative care). The availability of PGHD during in-person consultations could facilitate more objectively grounded discussions between HCPs and patients. It could help to give HCPs a clearer picture of a patient’s condition, improving the SDM process [7,19]. HCPs also believe that the integration of PGHD could improve symptom monitoring and assessment between visits, help personalize interventions and monitoring plans, aid with assessing clinical outcomes, promote patient self-management and behavior change support and prevention, as well as improve care delivery and quality assurance [22].

Motivated by the vision that PGHD are believed to help HCP “fill in the gaps” [26] and to unlock their potential, national frameworks have also been developed which guide their integration and governance. For example, the European Health Data Space [27], Digital Health Applications [28], and the UK’s Personalized Health and Care 2020 framework [29] are frameworks or legislation that support the use, governance, interoperability, and integration of PGHD, fostering the uptake of digital health technologies by patients and HCPs alike. However, there are several technical, social, and organizational challenges to integrating PGHD in clinical practice [7,12].

From an HCP’s perspective, PGHD can be challenging to use unless optimally prepared due to time constraints during consultations [12,30,31], the presence of irrelevant information or data, unfamiliarity with the structure of the collected data [32], and poor integration of PGHD into EHR systems [9], clinical workflows, and procedures. Furthermore, overheads due to administrative tasks related to data processing have been shown to create unnecessary workload and even burnout among health care providers [33].

From a patient perspective, there have been usability issues with the technologies, a shift in patient autonomy [31], increased occurrences of over-engagement with data which could in turn lead to a broad range of issues, including anxiety or depression and increased burden of tracking [9]. Moreover, patient expectations of HCPs to review their PGHD in its entirety during a time-limited consultation [34], emotional attachment and subjective view of their PGHD, and selective disclosure of PGHD could prevent doctors from having an unbiased view of patients’ data [21].

Figueiredo and Chen [35] systematically explored the various dimensions of PGHD (eg, type, intended use, and duration) and highlighted the critical need to consider sociotechnical factors for its successful integration. This aligns with the broader shifts in health care, including the rise of information infrastructures in health care [36] and increased *data work* required of both patients and HCPs. The HCI community has also embarked on efforts to have a deeper understanding of this phenomenon [37,38]. PGHD integration should seek to balance the needs and interests of both patients and HCPs, as PGHD “negotiation” [21] between them may increase tensions and “asymmetries” [6] between them. Such tensions underscore the importance of designing systems that appropriately mediate the interaction between patients and HCPs, thereby enabling effective patient-centered care and SDM.

#### Designing for PGHD Integration and Patient-HCP Collaboration

Past works show that the generation of PGHD is not performed at a single point in time. Two seminal works evaluate this from a systematic perspective, though not specifically from the patient’s viewpoint. Li et al [20] derived a stage-based model of personal informatics systems with five stages, namely (1) data preparation before tracking, (2) collection of information about oneself, (3) integration of information for sensemaking, (4) reflection on their personal information, and (5) taking action based on the information reflected upon. Primarily targeted toward the “quantifiers,” it emphasizes the need for personal informatics systems to be designed in a “holistic manner across the stages.” Epstein et al [39] subsequently adopted a *lived informatics* [40] perspective to characterize the integration of self-tracking into a quantifier’s daily life. While these models primarily focus on personal use by “quantifiers,” their stages and concepts can also be applied to patients who generate and share their data during clinical consultations. To systematically address the barriers HCPs face when integrating PGHD, West et al [12] developed a 6-stage workflow for integrating PGHD in clinical settings. The six stages are, namely, (1) aligning patient and HCP objectives, (2) evaluating data quality, (3) judging data quality, (4) rearranging the data, (5) interpreting the data, and (6) deciding on a plan or action. Overall, there needs to be alignment between HCPs and patients to pave the path toward patient-centered care.

Andersen et al [41] describe how patients and HCPs might have varying concerns in a clinical setting and emphasize that it is a challenge to align their concerns. They explain that for HCPs, concerns are about “professional issues to do with diagnosing and curing disease in accordance with accepted medical standards.” Whereas for a patient, concerns typically are about “personal experience and quality of life issues.” They introduce a set of concepts for analyzing concerns that arise during the design of e-Health systems, those being (1) meaningful and making sense to both HCPs and patients, (2) actionable, wherein either HCPs or patients can act on it, and (3) feasible, wherein it is easy and convenient to do so within the organizational and social context [41]. Adopting the concepts by Andersen et al [41] for designing e-Health systems, we posit that the PGHD collected by patients must be (1) meaningful through structured scaffolding of how and what data is collected and presented; (2) actionable, where both HCPs and patients could craft and adopt appropriate (and possibly personalized) treatment plans; and (3) feasible where in-depth user-centered design methods could be used to understand how different hospital systems work, HCPs’ practices, and the variances in sociocultural practices around patients’ collection and sharing of PGHD among different geographic locations. To effectively integrate PGHD into patient data journeys, it is essential to adopt a systemic lens to this challenge and to address the needs of the adopters [42].

#### Research Gap and Motivation

Over the years, several publications have investigated the integration of PGHD into clinical practice from the patients’ or the HCPs’ perspectives [7,21,31,43-48]. To date, a summary of the enablers and barriers to using PGHD from both perspectives has not been published yet. In a study on patient-provider collaboration in the context of irritable bowel syndrome and weight management, Chung et al [21] emphasize that collaboration occurs in every stage of self-tracking and suggest that due to the social nature of patient-provider collaboration with data, it is necessary to investigate how HCPs and patients’ roles shift across the stages. We aim to address this gap and align patient and HCP “concerns” [41] for better integration of PGHD in patient data journeys and clinical workflows.

## Methods

### Study Design

This scoping review is reported following the PRISMA-ScR (Preferred Reporting Items for Systematic Reviews and Meta-Analyses extension for Scoping Reviews) [47]. The scoping review approach was chosen over a systematic review process [49] to determine coverage of literature on the topic of PGHD integration in clinical pathways from the perspectives of both HCPs and patients, with an aim of aligning their needs. Prior reviews [12,25,50,51] around PGHD have focused primarily on a single stakeholder—HCPs. A formal review protocol was not registered or made publicly available.

We used the methodology formulated by Peters et al [52] to investigate the following research question: What barriers and enablers exist among key stakeholders—HCPs and patients—to integrate PGHD to inform SDM*?*

### Search Strategy

#### Overview

After reviewing prior works in this area and having a consultation among the coauthors, we narrowed down to ACM Digital Library, PubMed, and IEEE databases, which cover research in both technical and medical fields. The search was conducted in PubMed, ACM Digital Library, and IEEE Xplore, chosen to capture literature spanning clinical, technical, and human-computer interaction domains. The search was conducted in March 2023 for papers published between March 2013 and March 2023. We chose 2013 as the starting year as it was a crucial point in the evolution of the field of digital health technologies driven by the democratization of smartphones and wearables [53]. Furthermore, in 2013, acknowledging this phenomenon, the FDA released its first clear framework on how digital health apps and wearables integrations would be regulated for personal and clinical use [54].

Several keywords were initially selected after considering highly visible related works in the area. Thereafter, the first and second authors discussed common themes to narrow down to the keywords in a scheme that can be summarized as follows:

1. patient-generated data *OR* patient-generated health data *OR* [...]
  AND
2. patient-reported outcomes *OR* self-tracking *OR* digital health
  AND
3. shared decision-making *OR* [...]
  AND
4. enablers *OR* barriers OR perspectives OR [...]

As the terminologies patient-generated data and PGHD have been used in prior work, both keywords were included in the search query. Other related keywords indicated as “[...] ” and Boolean operators were included in the entire search query, which can be found in Multimedia Appendix 1.

#### Inclusion and Exclusion Criteria

The aim of applying inclusion criteria (ICs) and exclusion criteria (ECs) is to extract only publications relevant to the objective of this scoping review. We used the following set of ICs: (1) papers published in English in conference proceedings or a peer-reviewed journal, (2) papers related to noninvasive wearables that generated PGHD (eg, smartwatch, blood pressure monitor, etc) and patient self-reported data via journaling, etc, (3) studies which involved either HCPs or patients or both, (4) studies that explicitly mention SDM in person or remotely, mediated via technology, or have elements of SDM, and (5) the type of self-tracking could either be patient- or participant-initiated or clinician-initiated.

We used the following set of ECs: (1) review papers and study protocols, (2) papers referring solely to the personal health record of the patient, (3) papers with implants or wearables that require invasive surgery, and (4) gray literature and policy documents.

Further details about the IC and EC can be found in Multimedia Appendix 2.

#### Screening Process

To streamline the paper selection and review process, the results were exported to an AI-assisted collaborative review tool, Rayyan, which aids in duplicate detection and scoring suggestions. The AI system learns the patterns in the decision criteria and gives scoring suggestions after the author makes at least 50 inclusion or exclusion decisions. The first and second authors independently performed abstract screening and review of papers, and conflicts were resolved through discussions between the first and the second author after considering the research protocol and research question. The first author subsequently extracted the data from the final selection of papers. The detailed procedure is reported in the Results section.

#### Collating and Summarizing the Data

The extracted data were analyzed using a hybrid deductive-inductive thematic analysis guided by the 6-phase framework by Braun and Clarke [32] and the integrated approach to hybrid coding outline by Fereday and Muir-Cochrane [55] by a single researcher (see positionality statement in next subsection). The 6-stage workflow model initially proposed by West et al [12] was used as an a priori deductive framework. Initial data handling and preliminary inductive coding were performed by the first author using Excel (Microsoft Corp). To facilitate systematic analysis, the data and initial codes were then imported into MAXQDA (version 22). The first author conducted another round of familiarization to immerse in the data to refine the codes after consultation with the second author. Concurrently, open inductive coding was applied to capture barriers and enablers that fell outside the predefined framework. To ensure conceptual alignment, after reviewing the first 10 papers, the first author discussed code discrepancies with the second and last author. After coding for all the papers, the first, second, and last authors discussed the final codes and discussed the emerging themes. Clinician was used as shorthand for a wider understanding of HCP in this step. The detailed subthemes and corresponding categorization can be found in the codebook in Multimedia Appendix 3. We did not perform a formal critical appraisal of the papers, as the aim of this scoping review was to map the breadth of barriers and enablers reported across diverse study designs rather than to estimate any effect sizes.

### Positionality

The lead researcher is a PhD candidate with a background in human-computer interaction and digital accessibility and has industry experience in digital transformation and building systems for Digital Health Interventions. He comes from a highly digitized nation in Southeast Asia with a robust health care system leading in digital health. His PhD research agenda focuses on PGHD integration in cardiac prevention pathways, and as such, he has vested interests in understanding the sociotechnical factors influencing its integration. To manage potential biases in single-coder analysis, he kept notes of his coding process and discussed emerging codes and themes with the second and last author. The authors have a background in the field of human-computer interaction, digital health, health informatics, cardiology, and digital health interventions for physical activity behavior change and human-computer interaction.

## Results

### Identified Papers

**Figure 2.**
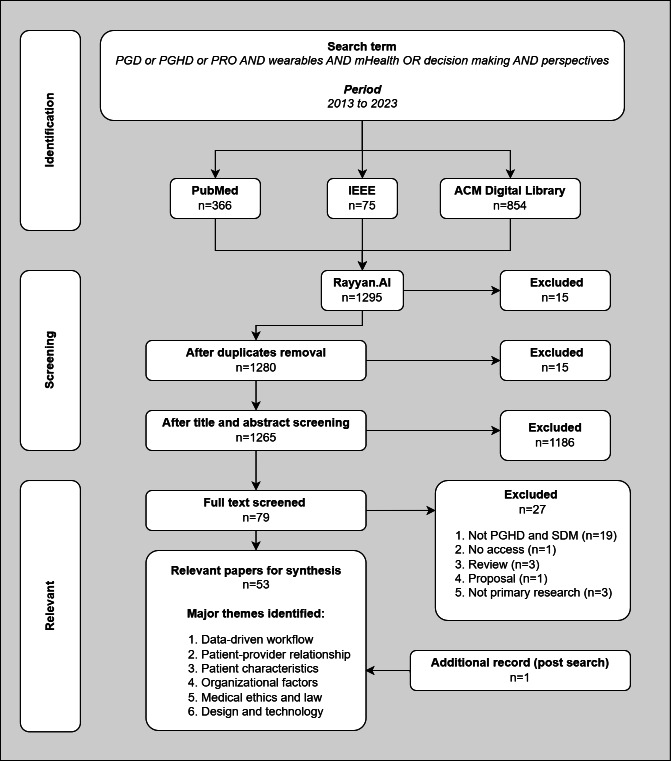
Flow diagram showing data extraction process. mHealth: mobile health; PGD: patient-generated data; PGHD: patient-generated health data; PRO: patient-reported outcome; SDM: shared decision-making.

Our search in the ACM, PubMed, and IEEE digital libraries resulted in 1295 records. The distribution of the results was as follows: ACM 854, PubMed 366, and IEEE 75. After the automatic removal of duplicates by Rayyan and manual removal by the first author, 1280 papers remained. Next, the first and second authors read all titles and abstracts to filter by IC and EC, and 1186 papers were removed, resulting in 79 papers. After full-text screening, 27 papers were removed. Finally, 52 papers were selected for the full-text review by the first two authors. The first author subsequently performed the data extraction using a predefined Excel sheet that captured study characteristics and stakeholder-reported barriers and enablers. One paper was manually added as it was mentioned as an influential related work in four papers [34,44,47,56] published in subsequent years, resulting in 53 papers [6,7,9,12,13,21,30,31,34,43-48,56-93] (see Multimedia Appendix 4). A validation check-in was performed with the last author after reviewing 25 papers. The flow diagram for the review is provided in Figure 2.

### Characteristics of Included Papers

The works predominantly originated from English-speaking countries (Table 1). About 50% (n=33) of them were from the United States and more than 10% (n=7) from the United Kingdom. The final number is higher than in 53 studies, as some studies were conducted in more than one country.

**Table 1.** Number of included studies by country of study setting.

Country/region	Values, n (%)
United States	33 (53.2)
United Kingdom	7 (11.3)
South Korea	3 (4.8)
Netherlands	3 (4.8)
Italy	2 (3.2)
India	2 (3.2)
Germany	2 (3.2)
Canada	2 (3.2)
Japan	1 (1.6)
France	1 (1.6)
Denmark	1 (1.6)
China	1 (1.6)
Brazil	1 (1.6)
Belgium	1 (1.6)
Australia	1 (1.6)
European Union	1 (1.6)
Total	62 (100)

**Figure 3.**
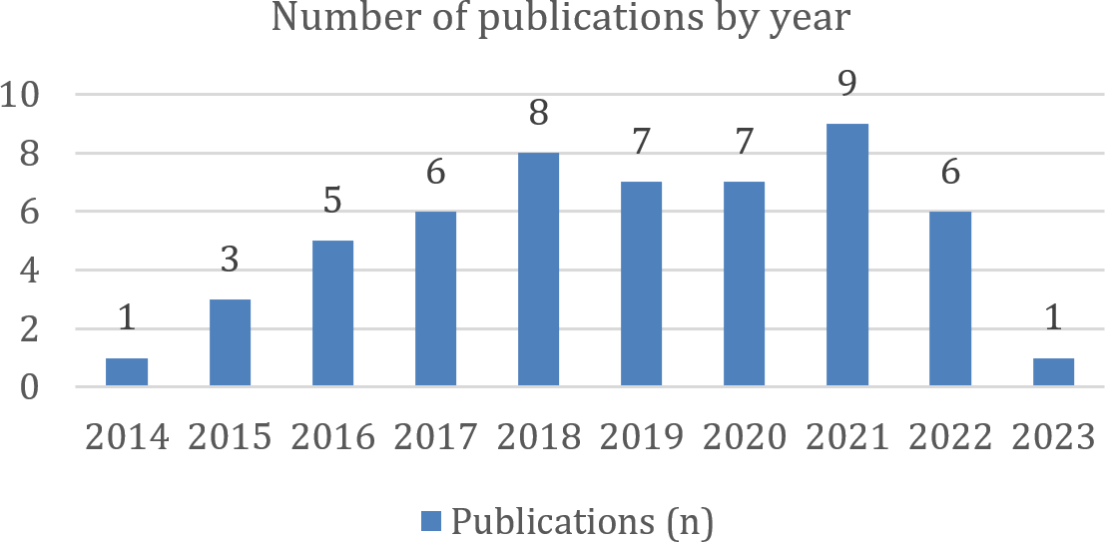
Number of publications per year (March 2013 to March 2023).

The number of papers published annually (Figure 3) has steadily climbed and peaked in 2021. The drop in 2022 could be due to the COVID-19 pandemic, which resulted in a decline in in-person consultations. Furthermore, as the search was conducted in the first quarter of 2023, this resulted in a low count. Most of the papers were about noncommunicable diseases and chronic conditions. Only one paper included perspectives of patients with communicable diseases [91].

### Factors Affecting the Integration of PGHD for SDM

In this section, we present the findings related to our guiding research question. The analysis identified several enablers and barriers related to the following six themes: (1) patient-provider collaboration with data, which refers to the interaction between HCPs and patients and several factors that affect this interaction, such as trust, expectations over data use, etc; (2) patient characteristics were mainly attributed to factors influencing patient behaviors and tracking habits or the effects of tracking; (3) organizational factors were related to processes within health care systems that could be improved or existing barriers preventing PGHD from integrating effectively into clinical workflows; (4) medical ethics and law refer to ethical and legal considerations such as patient autonomy, the use of PGHD for insurance purposes, and the questionable value of PGHD for clinical decisions; (5) data-driven workflow-related factors are broadly associated with characteristics, processes, and tools related to data, such as integration, standardization, relevance, trustworthiness, etc; and finally, (6) design and technology refer to the technical implementation of tools that generate or use PGHD and the consideration of human factors in such tools.

**Figure 4.**
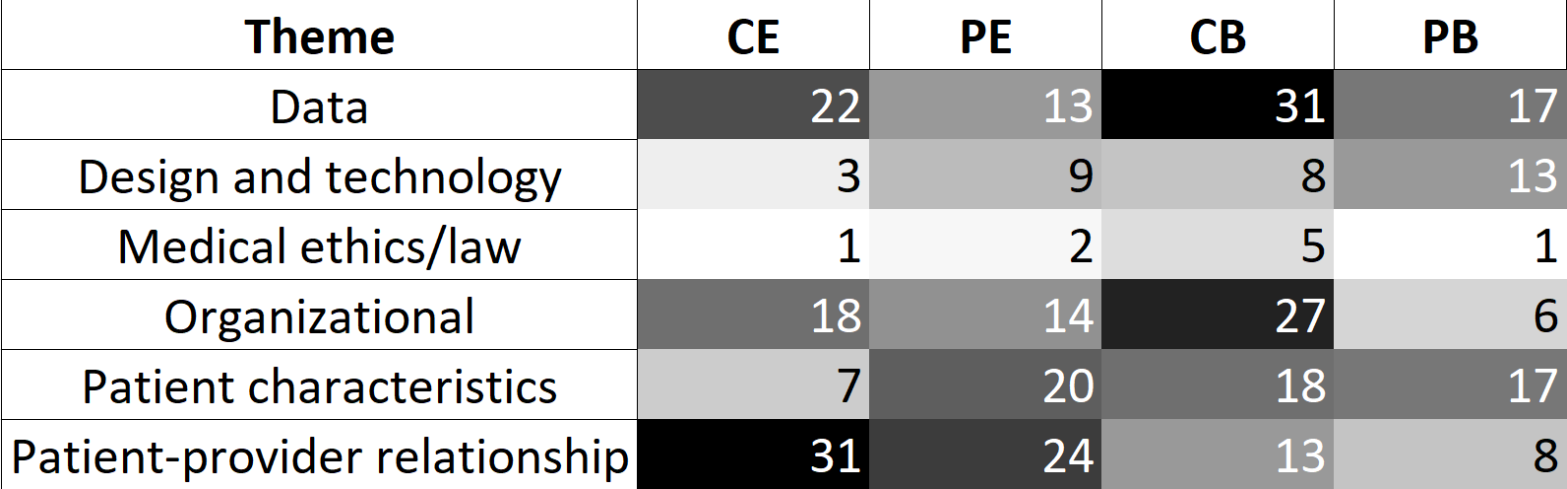
Heatmap showing the distribution of themes across stakeholder categories. Counts represent the number of studies in which a theme was identified at least once. A darker shade represents a higher count. CB: health care professional barrier; CE: health care professional enabler; PB: patient barrier; PE: patient enabler.

**Figure 5.**
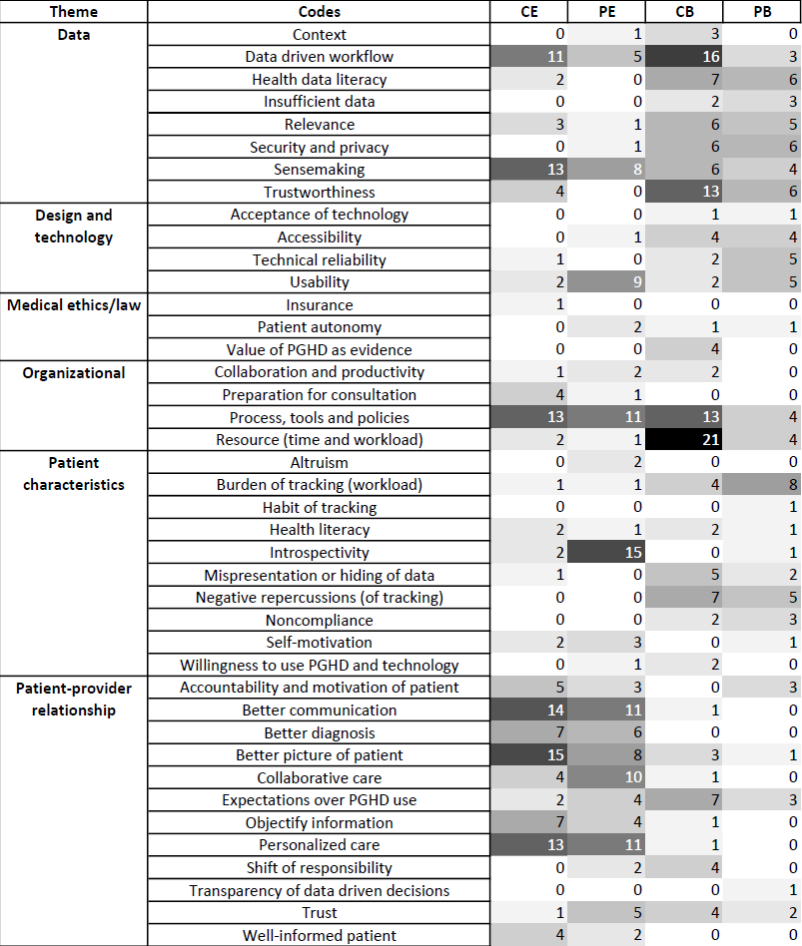
Heatmap showing the distribution of high-level codes representing barriers and enablers across stakeholder categories. Counts represent the number of studies in which a code was identified at least once. A darker shade represents a higher count. Multiple coded segments within the same study were counted as a single occurrence. CB: health care professional barrier; CE: health care professional enabler; PB: patient barrier; PE: patient enabler; PGHD: patient-generated health data.

The heatmap (Figure 4) shows the breakdown of themes by barriers and enablers and stakeholders based on the number of occurrences throughout the 53 papers selected for this review. Multiple coded segments within the same study were counted as a single occurrence. We also present a detailed heatmap showing the breakdown of codes by barriers and enablers raised by HCPs and patients in Figure 5. In Multimedia Appendix 5, we present an Excel sheet with detailed mapping of clinician or patient enablers or barriers across the 53 papers.

The most significant enabler for PGHD integration is how it could promote patient-provider collaboration with data, especially concerning improving communication, allowing for patient-centered care, and providing HCPs with a better picture of the patients. Aspirations followed this in terms of having mechanisms that enable effective sensemaking of health data and having data-driven clinical workflows.

Among barriers, data-driven workflow (eg, lack of standards, poor integration, and lack of access to data) has emerged as the most significant, followed by organizational factors (eg, unavailability of tools and lack of time) and patient characteristics (eg, selective disclosure of data and increased burden of tracking). In the subsequent sections, we will delve deeper into the specific factors.

### Patient-Provider Collaboration With Data

Patient-provider collaboration with data is mainly seen as an enabler for the integration of PGHD. The key enablers include the ability to offer personalized care, facilitate communication between patients and HCPs, and improve diagnosis. For example, “both healthcare providers and patients use data to approach the unknown, whether they use it to identify the possible causes of infertility, to identify the fertile window, to decide on potential treatments, or to choose follow-up treatments” [56]. PGHD is also seen as an aid in building trust by objectifying subjective information: the physiotherapists found that the information provided by FitViz helped them in conducting *“*patient-driven” consultations [66].

While patients are often enthusiastic about sharing the data with HCPs, “patients also wanted to use their data to get recognition for their efforts and to show their doctor they take their health plan seriously” [21]. HCPs are concerned about a shift of responsibility as they might be more accountable for the data shared by the patients: “clinicians were concerned that by having continuous access to patient data, they would become responsible for monitoring that data for concerning changes that may be indicative of a mental health crisis, such as a manic episode or a suicide attempt” [65] and feel an obligation to interpret and explain the data: “simply” receiving PGHD around daily symptoms created a feeling of an ‘obligation to act’ for the clinicians” [71]. Moreover, with the capability of remote monitoring, patients “want to have the ability for their physician to conduct remote monitoring to verify the treatment plan is working and to pick up early warning signs of relapse or deterioration” [48].

### Patient Characteristics and Perceptions

HCPs, in general, are worried about the (unintended) consequences of patients tracking and bringing their PGHD to consultations. They feel that an increased emotional attachment of patients to their PGHD could lead to depression and anxiety. For example, therapists highlight that “depression could be a potential side effect of tracking” [84] and patients could also “overreact to [negative] data” [43]. Both HCPs and patients have concerns about the effort required for tracking and feel that tracking could increase patients’ burden; in a study by Ancker et al [91], a diabetes patient who had given up self-monitoring of blood glucose said, “it’s too cumbersome for me.”

In addition, patient motivations for sharing data with HCPs are not always clear due to *selective disclosure,* as the “patient makes the decision about what kind of data the clinician can see. [As a result] clinicians [were] uncomfortable” [65] with such data unless they had the entire dataset. HCPs are concerned about the “increased transparency via PGHD and how it could lead to noncompliance [by patients]*”* [31].

Although most feedback was negative, HCPs feel that having access to data could allow patients to better engage with their data through *introspection* and *motivate* them to self-treat their conditions. Patients feel more self-aware of their condition, and self-tracked data helps them see their habits [21]. Furthermore, patients also believed that sharing their data could benefit future research for treating patients with similar conditions: “they hoped their Fitbit data would benefit future veterans” [78].

### Data-Driven Workflow

The data-driven workflow theme comprised several enablers and barriers. Within the theme, there are several factors considered as enablers, among which the generation of data, data actionability, and having access to standardized tools integrated well into the clinical workflow.

From the HCP’s perspective, data sensemaking is a key enabler for unlocking the value of PGHD. For example, HCPs feel that “when patients struggled to identify triggers, visualizations of quantitative analyses could help patients and providers understand underlying nutrient-symptom relationships” [31]. Data sensemaking enabled patients to effectively use PGHD, facilitating better reflection of their health conditions and empowerment of self-care. For example, in a study of diet monitoring, “HCPs and participants were able to [make sense of their data to] identify eating patterns or potential triggers and used Foodprint as a structuring artifact to discuss actionable next steps” [34]. However, in a study with diabetes patients and providers, “commercially available visualizations that are built using clinical guidelines were useful for providers but not for patients” [83]. HCPs want standardized tools to interact with data and better understand the patient’s condition. For instance, HCPs stress the importance of a “uniform clinician interface that integrates [PGHD] from various individual tools, supporting the findings in previous studies” [85] and take appropriate action, leading to better data actionability. Contextual information—how and when the data was collected—is also mentioned as a key to data actionability. Access to the data’s context could allow for the discovery of “possible confounds including emotional and physical health, hydration, and exercise” [82]. Integration could enable HCPs to *access* and review data before consultations. In one of the studies, *“*geriatricians preferred to review the data before the consultation, and then implement a new part of their care plan based on the data” [70].

There are also several barriers identified. The introduction of new tools and technology meant that HCPs must have the necessary *literacy for* interacting with data. Some “[HCPs] doubt their ability to advise on tracking; many providers doubt patient ability to review tracking data” [30]. HCPs also highlight the need for better *data integration* with existing EHR and electronic medical records systems. For example, Zhu et al [81] found that as “commercial sleep tracking technology did not support data exporting or sharing, many clinicians continued to use paper sleep diary.”

HCPs were also concerned about PGHD’s *quantity*, *quality*, and *reliability*, which could, in turn, affect its *trustworthiness*. When “different patients use different apps, it [became] difficult for health care providers to interpret the data and assess the reliability of each app” [58]. Regarding the amount of data, some HCPs’ feedback about receiving or reviewing data relevant to the consultation, given that unnecessary data could result in ineffective decision-making. From the patient’s perspective, there is also a lack of awareness about what to track. As a result, *“*many wanted to track more data than may be feasible*”* and wanted to have *“*smart defaults for how to track because they did not know what would reasonably balance burden and usefulness*”* [46].

### Organizational Factors

Organizational factors mainly encompass factors about a health care organization such as a hospital. The primary barrier is HCPs’ lack of time—“the cardiologists in our sample already encounter problems with data overload in their daily practice” [80] and the need for more time and effort to review patient data. HCPs share that “patient-provided data sources will add layers of data assessment to practice, and questioned whether this information may adversely affect efficient workflow” [87].

HCPs need more support, tools, and incentives from upper management to integrate PGHD into clinical care practices. HCPs feel that although activity monitoring tools could help to streamline means of collecting data, “current activity monitoring tools are insufficient to address program specific prehabilitation assessment needs; to exacerbate this challenge, stakeholder tailored tools do not currently exist” [67]. Regarding clinical workflow, HCPs feel that the ready availability of PGHD before consultations could be beneficial in allowing them to review patients’ conduct to *prepare for consultations* beforehand, leading to better consultations.

*Nearly all providers agreed that the best time to receive the patient-generated health report was immediately before a scheduled clinic visit*.

[92]

This, in turn, leads to better consultations: “pre-visit notes helped health experts focus on participant goals and questions when reviewing food photos” [34].

### Medical Ethics and Legal Aspects

Medical ethics and legal aspects refer to the responsibility of HCPs, ethics, reimbursement, and legal aspects of data. HCPs are worried about regulations such as HIPAA (Health Insurance Portability and Accountability Act) obstructing the introduction of PGHD into their care practices. Furthermore, autonomy is a factor for concern among HCPs and patients. For example, some “older adults perceived that monitoring PGHD increased the transparency of their (lack of) engagement in healthy behaviors” and “perceived [it] as a threat to autonomy” [31]. Finally, the unproven value of PGHD in health care—“lack of evidence for use” [9] of PGHD raises ethical concerns about how such data can be used for clinical decision-making, thereby limiting its adoption. An HCP felt frustrated rearranging the PGHD data and structuring it as evidence within their clinical framework [87]. Within the broader theme of ethics and legalities, one HCP mentions how PGHD could benefit insurance concerning a patient’s recovery: “I think it’s also good to show insurance kinda, hey, we are making changes, you know, getting them better in this aspect” [84].

### Design and Technology

Both HCPs and patients highlight that well-implemented technologies with good usability can be a driver for adoption and sustained use. For example, among patients, learnability and ease of use—subqualities of usability—are favorable for patients in two studies: “GeniAuti made their recording process easier in everyday life” [60] and “ease-of-use of DiaFocus and found it easy to use and easy to learn to use” [57].

For HCPs, the usability of their tools is a key factor for adopting PGHD and missing features such as “inability to sort the data” [89] and added additional time. HCPs are also concerned about the reliability of the applications and the technologies that the patients adopted, especially when patients might use a myriad of them based on their preferences. On the other hand, “when hospitals offer the same app that has been clinically validated to all patients, health care providers can become, over time, knowledgeable about [it]” [58].

Overall, both stakeholders believe that technologies should be designed to be accessible so that population groups such as older adults can adopt these technologies.

*Clinicians also alluded to old age as a potential challenge as the older generation is not as fluent with mHealth, wearable technology, and other devices that collect PGHD*.

[31]

In a particular study, a participant’s medical condition—rheumatoid arthritis—caused issues with their participation due to the tool’s [lack of] accessibility [63].

### PGHD Across a Patient Data Journey and HCP’s Clinical Workflow: 10-Stage Workflow

Our analysis includes a wide range of barriers and enablers which can be arranged across a patient’s journey and HCP’s clinical workflow to provide logical flow. In doing so, we extend and augment the work by West et al [12]—a 6-stage workflow model aligning patient and HCP objectives—and the work by Li et al [20]—the stage-based model of personal informatics systems—to demonstrate how PGHD is integrated beyond clinical settings (Figure 1). Our updated workflow identifies four new stages encapsulated within the pre- and postconsultation phases in addition to the existing stages identified by West et al [12]—stage (1) align patient and HCP objectives, stage (2) evaluate data quality, stage (3) judge data quality, stage (4) rearrange the data, stage (5) cointerpret the data, and stage (6) decide on a plan or action. It also emphasizes the dyadic relationship between the HCPs and patients and the numerous factors influencing the integration of PGHD along the patient journey and HCP’s clinical workflow. It starts from the data collection (stage 1) stage till a clinical action is taken (stage 10) and repeats (especially for patients with chronic or multiple conditions). The following subsections describe the newly added steps in further detail.

### Collect Data (Stage 1)

Data collection is a crucial stage where patients initiate the tracking of PGHD. In some cases, they might be embarking on this activity on their own or through social influence; on the other hand, it could have been due to instructions from their HCP, for example, when treating medical conditions over the long term. However, it is also worth noting that the monitoring process requires additional effort—although there are methods for passive collection, for example, through wearable or stationary sensing devices, these do require considerable effort to set up and maintain. As such, both active (eg, through occasional questionnaires or more frequent ecological momentary assessment) as well as passive sensing introduce an additional burden to patients—on top of managing their medical condition.

*[Patients] mentioned that daily measurements were too burdensome or medicalizing, especially when they perceived their symptoms or blood pressure to be stable.*

[69]

### Reflect on Data (Stage 2)

In the reflection stage, patients can interact with the data they collected through the various sensemaking mechanisms available. This could be presented as data summaries, PDF reports, or graphs of different formats. However, at this stage, there are both facilitators and barriers. By accessing such data, patients could reflect on their physiological data such as stress levels, weight, etc, and information such as physical activity trends.

*[P]atients looked to their data for self-awareness of their current lifestyle and described its value in terms of helping them see their habits*.

[21]

This could allow them to be well-informed about themselves before consultations. However, if “veterans do not understand or trust the data, it could be difficult for them to be motivated to use it” [78]. There were also incidents in specific medical conditions—for example, in mental health—where having access to such data could reinforce negative views of their condition and agitate symptoms [72] or demotivate patients. Additionally, the constant recurrence of a condition—hence having increased tracking burden—also prevented some patients from self-monitoring: “many unengaged patients reported being easily discouraged by their [arterial fibrillation] AF recurrence, which they said caused them to self-monitor less” [77].

### Integrate Data (Stage 3)

The integrate data stage entails the import of data into a clinical setting. Here, the patient attempts to “upload” their data into clinical systems, or the HCP could manually associate them with their EHR systems (a common need to store the foundations for decision-making). However, at this stage, HCPs faced trouble importing such data due to incompatibilities or insufficient interfaces to their systems. In a study, although “patients used mobile apps for their sleep diary, clinicians who received this data had to manually input each data point” [81] due to limitations in data sharing functionalities. There were issues about poor interoperability of self-tracking technologies—primarily due to the nonstandardization of devices or technologies—which prevented seamless integration into EHR systems. Notably, current-generation digital technologies for supporting sensemaking from PGHD cannot easily scale novel data types, handle incomplete or incongruent data, or integrate multimodal PGHD streams.

### Take Action (10)

Finally, in the *taking action* stage, after a consultation with an HCP, the presence of data could allow patients to be more accountable for self-treating their conditions based on decisions made during the consultations. However, in some cases, there was a concern about the HCP’s ability to monitor patients’ conditions remotely or with more granular details; this could invade patients’ privacy and threaten their *autonomy* [31]. In addition to this, although HCPs could give recommendations to patients on what tools to use to control their condition, they might not necessarily comply with it:

*Providers sometimes recommended tracking tools to patients for clinical diagnosis and management. However, patients do not always follow those recommendations.*

[13]

### Tensions Between HCPs and Patients

Having mapped the barriers and enablers across the 10 stages, we now turn to the cross-cutting tensions that emerged between HCP and patient perspectives throughout the workflow. Excerpts from relevant papers are presented below each point:

Regarding tension 1, patients need clarification or are unaware of the *relevance of data* and need more *health literacy*. This results in (*time-limited*) HCPs facing an overload of (unnecessary) data.

*[There is] a need to negotiate with patients when determining which data elements to collect, set patients’ expectations for communication about PGHD, and let patients know that they would not be contacted if everything was normal.*

[89]

*Older adults and clinicians perceived that the lack of clinical knowledge by patients leads to a collection of irrelevant PGHD and decreases the usefulness of the information.*

[31]

Regarding tension 2**,** patients, at times, have a subjective and emotional connection with their data and were hoping for detailed explanations of the data they brought for consultations. HCPs, however, intend to view the data objectively while making clinical decisions. This can result in mismatched expectations over collected data by patients and sometimes misunderstandings over how HCPs made clinical decisions using the data, which results in ineffective consultations.

*Patients also expected providers to engage with the data and provide a personalized treatment plan.*

[21]

*The conflicting views often led to disagreement between the caregivers’ and experts’ interpretations of the children’s challenging behaviors*.

[60]

Regarding tension 3, patients feel that increased monitoring and visibility of PGHD could lead to disruption of their autonomy and loss of privacy; however, HCPs feel that having (access to) more (readily available) data could allow for better health support.

*Participants were concerned about talking to clinicians they needed to become more familiar with and about the privacy of their PGD.*

[68]

Regarding tension 4, HCPs desire a *complete picture of the patients (to facilitate decision-making*) but at the same time feel challenged by *increased workload* due to the increased data and insufficient time to handle them.

*Provide the Context, Longer View, and Whole Picture.*

[90]

*If patients share the collected data with their doctor, the interpretation of the data and appropriate response becomes the doctor’s response.*

[80]

Regarding tension 5, patients have issues trusting the HCP’s ability to interpret and review data. Furthermore, HCPs themselves had self-doubts about their capabilities.

*These barriers include a lack of time to review detailed records, questions about providers’ expertise, and skepticism about additional benefits of reviewing data.*

[30]

*The visualizations also caused some unease for [participants], who mentioned feeling “embarrassed” or “anxious” about giving the impression that they lacked necessary knowledge to explain the visualizations.*

[82]

Regarding tension 6, patients and HCPs have varied preferences on which type of problem to focus on and how they wanted the data to be represented.

*Patients and providers differ on the type of problem to focus on.*

[83]

*Data gives different insights to patients and providers.*

[83]

*Patients and providers use different representations of data to identify problems.*

[83]

Regarding tension 7, ethical and legal challenges of data privacy, patient autonomy, and HCP accountability.

*...the use of mHealth contributed to them being in control of their own health and to bring their own perspectives to the fore in consultations with health care professionals.*

[69]

*Older adults felt that monitoring PGHD increased the transparency of their (lack of) engagement in healthy behaviors.*

[31]

## Discussion

### Summary of Results and Implications

Across the 53 reviewed papers, we identified 6 overarching themes influencing the integration of PGHD into patient journeys and clinical workflows to enable SDM: patient-provider collaboration, patient characteristics and perceptions, organizational factors, medical ethics and law, data-driven workflow factors, and design and technology. Furthermore, we surface 7 cross-cutting tensions faced by HCPs and patients. These findings are relevant for system designers and health care practitioners seeking to digitalize clinical pathways through the integration of PGHD.

Despite the potential of PGHD, there are still significant challenges. There is a lack of transparent processes, standardized tools, and communication about how data should be presented during consultations; individuals bringing their PGHD to consultations would currently most likely add additional workload for HCPs by providing irrelevant and nonstandard data and expecting interpretation. Furthermore, varying patient motivations and their selective data disclosure add layers of complexity to the relationship between HCPs and patients while integrating PGHD into clinical workflows.

Unpacking such challenges while making health and care increasingly patient-centric and assuring that patients feel heard and empowered is an essential goal in many national and international health care strategies (eg, other studies [94-97]). SDM is a key enabler of patient-centered care and helps with fostering adherence and self-determination as well as proactive approaches to health and care. In our 10-stage workflow model, we posit that stage 8 “cointerpretation of data”—or collaborative sensemaking between HCPs and patients—is the primary enabler for successful SDM when integrating PGHD. Prior work indicates that collaborative sensemaking of PGHD can improve patient-provider communication by facilitating deeper discussions regarding personal values and granting HCPs a more nuanced understanding of treatment progress [19,98]. As such, we argue that system designers should prioritize the development of interfaces that scaffold this collaborative sensemaking process to enable SDM.

### Toward Enabling Integration of PGHD in Patient Journey and Clinical Workflows

Information infrastructures in health care are embedded within social arrangements and technologies and are spatiotemporal in nature [36]. HCI has a central role to play in investigating them and creating health care technologies that are fit for purpose [17].

In a clinical context, HCPs may already be facing increased risk of burnout due to poor technology integration [33], and given the potential benefits and burden PGHD brings, there is a need to prioritize how data-related tools can be better integrated. On a higher level, there needs to be organizational support in creating policies and processes to support the use of PGHD and creating data-driven workflows that seamlessly integrate such data into clinical practices. However, we identified challenges in a clinical setting involving how patients collect data and collaborate with HCPs in a clinical context.

Work on aligning and interactively navigating priorities between HCP and patient [12,21] appears clearly implicated. Tensions could arise from patient preferences or the subjectivity of data due to the emotional connectivity of the data conflicting with HCPs adopting a very objective view of data with their medical background—typically due to medical ethics. By juxtaposing the needs and priorities of both stakeholders, we discussed the alignments and tensions between them. We also identified design opportunities for system designers to consider addressing and balancing the stakeholders’ priorities adequately by proactively building on enablers and avoiding or decisively tackling barriers.

Overall, the scoping review outcomes emphasize a need to adopt a systemic lens on the issue of integrating PGHD into clinical practice by considering its flow across the patient data journey and clinical workflows. Our research team, consisting of the first, second, and last authors, conducted a thorough review of the existing 6-stage workflow model [12] and the emerging codes to establish the new stages and the enablers and barriers at each stage, and the potential tensions. This process resulted in the augmented 10-stage workflow model (Figure 1). This model (see simplified model in Multimedia Appendix 6) includes pre- and postconsultation phases, as well as corroborated formerly identified barriers and enablers and newly identified ones.

### Addressing Barriers

To facilitate the practical application of our findings, we organize the barriers across the stages along with potential mitigation strategies in Table 2.

**Table 2.** Barriers and mitigation strategies.

Stages	Barriers	Mitigation strategies
Preconsultation		
Collect data (stage 1)	Burden of tracking	Use passive tracking as much as possible and recommend only tracking that is necessary for consultations.
Reflect on data (stage 2)	Negative repercussions of tracking	Educate patients on possible negative impacts of data and offer adequate resources to obtain support on data sensemaking.
Integrate data (stage 3)	Poor data integration	Standardize interfaces and data export formats among commercial devices and applications for better integration into EHR^a^ systems.
Post consultation		
Take action (stage 10)	Shift of patient autonomy and noncompliance	Ensure that patient preferences on how their data is viewed and accessed are respected and communicated with them transparently. Educate patients on the benefits of adherence.
All stages		
N/A^b^	Overburdened HCPs^c^	Across all stages: Implement data sensemaking support in-line with clinical workflows. Consider shifting to partially automated sensemaking steps that already occur before consultation
N/A	Lack of time for HCPs	Introducing tools to improve the operational efficiency of clinical workflows
N/A	Questionable value of PGHD^d^	Conduct empirical research on PGHDs and how they improve patient-centered care.

a EHR: electronic health record.

b N/A: not applicable.

c HCP: health care professional.

d PGHD: patient-generated health data.

### Addressing Tensions

Apart from addressing key barriers, it is essential to address divergent perspectives resulting in tensions between the stakeholders. Given the dyadic relationship between patients and HCPs during SDM, it is essential to understand potential misalignments between both stakeholders, which results in tensions. In Table 3, we delineate these specific tensions alongside targeted alignment strategies designed to harmonize stakeholder expectations and foster collaborative care.

**Table 3.** Tensions and alignment strategies.

Number	Tension	Alignment strategies
1	Patients’ lack of awareness about what data to bring vs time-starved HCPs^a^	Improve health (data) literacy of patients, align expectations, or establish standards over what data are needed for the consultation and for SDM^b^, thereby engendering mutual trust.
2	Patients’ subjectivity vs HCPs’ objectivity	HCPs should set expectations regarding how PGHD^c^ would be used for SDM and provide an explanation of how they influenced the SDM process.
3	HCPs’ need for access to patient data vs patients’ desire for privacy and autonomy	Patients and HCPs should set boundaries over how and when HCPs track PGHD to maintain patient autonomy. Appropriate privacy mechanisms could be designed for dynamic configuration of consent.
4	HCPs want to have a complete picture of patients vs limited consultation time	Equipping HCPs with the necessary tools such as clinical decision support and AI-assisted systems could help augment HCPs with PGHD interaction.
5	Patients’ perception of lack of HCP data literacy affected trust	Improve HCPs’ data literacy capabilities and assure patients about their abilities to make data-driven decisions.
6	Differing perspectives and approaches that patients and HCPs have when interacting with PGHD for diagnosis	Tools should promote mutual understanding between both patients and HCPs and support problem identification, preferably in an automated manner [83].
7	Ethical and legal need for addressing the dilemma of data privacy and patient autonomy vs HCP accountability	Foster cross-sectoral collaboration between patients, HCPs, and legislators to co-design the most appropriate governance frameworks for a given chronic disease context. This could include steps such as implementing dynamic consent and adapting policies such as HIPAA^d^ in the United States and EHDS^e^ to ensure that they do not diminish the quality of care [99]. Furthermore, alignment with medical ethics standards should incorporate national and contextual nuances, allowing for safeguards such as involvement of ethical committees for ethical oversight.

a HCP: health care professional.

b SDM: shared decision-making.

c PGHD: patient-generated health data.

d HIPAA: Health Insurance Portability and Accountability Act.

e EHDS: European Health Data Space.

Some of these alignment strategies could be considered as implications for design at an organizational or policy level based on the health care system. At an organizational level, system designers can adopt generative co-design activities with HCPs and patients, such as the approach by Höppchen et al [100] to design clinical pathways which systematically align the integration of PGHD.

### Limitations

Our scoping review’s findings were based on the papers derived from 3 databases, and other unpublished or nonpeer-reviewed works could be available in other repositories. They reflect studies primarily conducted in English-speaking, high-income countries, partly a result of limiting our search to English papers. As such, our review may underrepresent perspectives from low- and medium-income countries where infrastructural capabilities, sociocultural factors, and health care systems may vary significantly. We also acknowledge that no critical appraisal was conducted for individual studies as they are optional for scoping reviews and additionally due to the heterogeneity of the study designs included in our review. Furthermore, as mentioned in the positionality statement above, there could be researchers’ subjectivity in the interpretation of the findings. As HCPs and patients unequally contributed to the findings, the perspectives should be explicitly assessed in empirical work and in a balanced manner. However, these preliminary findings give readers and designers a sense of factors to consider when integrating PGHD into digital health technologies and wider health care systems.

### Conclusions

PGHD has immense potential to enhance patient-centered care and lead toward P4 (predictive, preventive, personalized, and participatory) medicine. However, its widespread integration in clinical settings still needs to be improved. Our research question aimed to paint a holistic and ecological understanding of the PGHD integration barriers and enablers perceived or experienced by patients and HCPs to better facilitate PGHD integration. By reviewing works from the past 10 years and incorporating both patient and HCP perspectives, we identified several enablers and barriers across 6 key themes. We build on the previous 6-stage model by West et al [12], adding 4 new stages beyond the clinical setting, including “collect data,” “reflect on data,” “integrate data,” and “take action,” and added the perspectives of both patients and HCPs to identify new barriers and enablers across the various stages of interaction with PGHD. Our 10-stage workflow model of aligning patient and HCP objectives in integrating PGHD implies several challenges to be tackled in a complex health data ecosystem [18] and health data lifecycle [17]. Furthermore, the model forms the foundation for deriving design implications around tensions and alignments between HCPs and patients along data-enabled patient journeys and clinical workflows. By addressing challenges introduced by PGHD, SDM could be more efficient for both parties, leading to better patient-centered care and life-accompanying personalized data-enabled health. Future work should investigate how our workflow model performs in real-world settings and expand evidence from underrepresented regions to improve the global applicability of PGHD integration among different health care systems.

## Supplementary material

10.2196/85197 Multimedia Appendix 1 Search query.

10.2196/85197 Multimedia Appendix 2 Inclusion and exclusion criteria.

10.2196/85197 Multimedia Appendix 3 Themes and definitions.

10.2196/85197 Multimedia Appendix 4 Characteristics of included papers charted as a table. The study ID listed in this document is to be used in conjunction with the spreadsheet in Multimedia Appendix 5.

10.2196/85197 Multimedia Appendix 5 An Excel sheet with detailed mapping of clinician or patient enablers or barriers across the 53 papers.

10.2196/85197 Multimedia Appendix 6 Simplified 10-stage workflow model for integrating patient-generated health data.

10.2196/85197 Checklist 1 PRISMA checklist.

## Abbreviations

EC: exclusion criterion
EHR: electronic health record
HCP: health care professional
HIPAA: Health Insurance Portability and Accountability Act
IC: inclusion criterion
P4: predictive, preventive, personalized, and participatory
PGHD: patient-generated health data
PRISMA-ScR: Preferred Reporting Items for Systematic Reviews and Meta-Analyses extension for Scoping Reviews
SDM: shared decision-making
