# Supplementary material for: Barriers and Enablers to Integrating Patient-Generated Health Data in Shared Decision-Making From Health Care Professional and Patient Perspectives: Scoping Review
Source: JMIR Mhealth Uhealth. 2026 Aug 10;14:e85197. doi: 10.2196/85197 (PMC13456309; doi:10.2196/85197)
Supplement: Supplementary file 2 [file mhealth-v14-e85197-s006.docx]

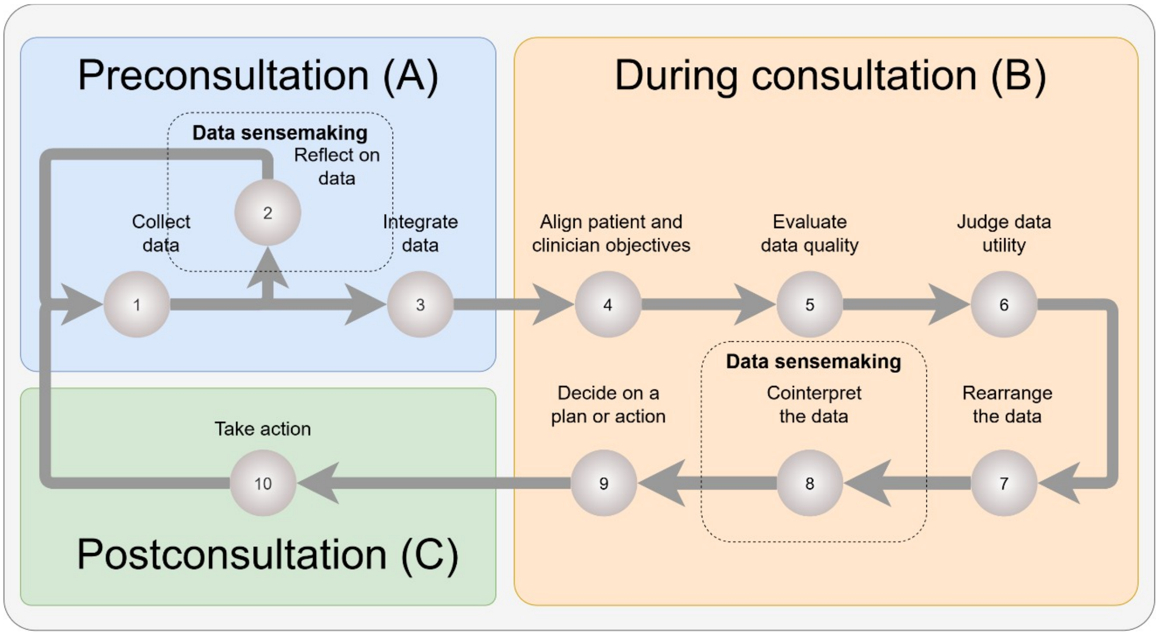
Preconsultation (A)
Data sensemaking
Reflect on data
2
Collect data
1
Integrate data
3
During consultation (B)
Align patient and clinician objectives
4
Evaluate data quality
5
Judge data utility
6
Data sensemaking
Decide on a plan or action
9
Cointerpret the data
8
Rearrange the data
7
Take action
10
Postconsultation (C)
