# Supplementary material for: Barriers and Enablers to Integrating Patient-Generated Health Data in Shared Decision-Making From Health Care Professional and Patient Perspectives: Scoping Review
Source: JMIR Mhealth Uhealth. 2026 Aug 10;14:e85197. doi: 10.2196/85197 (PMC13456309; doi:10.2196/85197)
Supplement: Supplementary file 2 [file mhealth-v14-e85197-s001.docx]

ACM Digital Library

"PGD" OR "PGHD" OR "Patient-Generated Data" OR "Patient Generated Data" OR "Patient Generated PGD" OR "Patient-Generated PGD" OR "personal health*" OR "personal informatic*" OR "patient-contributed data" OR "patient contributed data" OR "patient-centered data" OR "patient-centred data" OR "Patient Centered data" OR "Patient Centred data" OR "patient-reported outcomes" OR "patient reported outcomes" AND "self-track*" OR "self track*" OR "body-worn*" OR "body worn*" OR "wearable*" OR "smartphone*" AND "mhealth" OR "telehealth" OR "mobile health" OR "health" OR "medic*" OR "digital-health" OR "digital health" OR "shared decision-making" OR "shared decision making" OR "clinical decision-making" OR "clinical decision making"OR "SDM" OR "patient-provider collaboration" OR "patient provider collaboration" OR "doctor-patient communication" OR "doctor patient communication" OR "telerehab" OR "Telerehabilitation" AND"Barriers" OR "hurdles" OR "needs" OR "problems" OR "Facilitators" OR "enablers" OR "Opportunities" OR "Goals" OR "Aspirations" OR "Perspectives" OR "Experiences" OR "Perceive" OR "Experience" OR "Lessons"

PubMed

"PGD" OR "PGHD" OR "Patient-Generated Data" OR "Patient Generated Data" OR "Patient Generated PGD" OR "Patient-Generated PGD" OR "personal health*" OR "personal informatic*" OR "patient-contributed data" OR "patient contributed data" AND "self-track*" OR "patient-contributed data" OR "patient contributed data" OR "patient-centered data" OR "patient-centred data" OR "Patient Centered data" OR "Patient Centred data" OR "patient-reported outcomes" OR "patient reported outcomes" AND "self track*" OR "body-worn*" OR "body worn*" OR "wearable*" OR "smartphone*"AND"mhealth" OR "telehealth" OR "mobile health" OR "health" OR "medic*" OR "digital-health" OR "digital health" OR "shared decision-making" OR "shared decision making" OR "clinical decision-making" OR "clinical decision making"OR "SDM" OR "patient-provider collaboration" OR "patient provider collaboration" OR "doctor-patient communication" OR "doctor patient communication" OR "telerehab" OR "Telerehabilitation"AND"Barriers" OR "hurdles" OR "needs" OR "problems" OR "Facilitators" OR "enablers" OR "Opportunities" OR "Goals" OR "Aspirations" OR "Perspectives" OR "Experiences" OR "Perceive" OR "Experience" OR "Lessons"

IEEE

PGD" OR "PGHD" OR "Patient-Generated Data" OR "Patient Generated Data" OR "Patient Generated PGD" OR "Patient-Generated PGD" OR "personal health*" OR "personal informatic*" OR "patient-contributed data" OR "patient contributed data" OR "patient-contributed data" OR "patient contributed data" OR "patient-centered data" OR "patient-centred data" OR "Patient Centered data" OR "Patient Centred data" OR "patient-reported outcomes" OR "patient reported outcomes" AND "self-track*" OR "self track*" OR "body-worn*" OR "body worn*" OR "wearable*" OR "smartphone*"AND"mhealth" OR "telehealth" OR "mobile health" OR "health" OR "medic*" OR "digital-health" OR "digital health" OR "shared decision-making" OR "shared decision making" OR "clinical decision-making" OR "clinical decision making"OR "SDM" OR "patient-provider collaboration" OR "patient provider collaboration" OR "doctor-patient communication" OR "doctor patient communication" OR "telerehab" OR "Telerehabilitation"AND"Barriers" OR "hurdles" OR "needs" OR "problems" OR "Facilitators" OR "enablers" OR "Opportunities" OR "Goals" OR "Aspirations" OR "Perspectives" OR "Experiences" OR "Perceive" OR "Experience" OR "Lessons"
