# Supplementary material for: Barriers and Enablers to Integrating Patient-Generated Health Data in Shared Decision-Making From Health Care Professional and Patient Perspectives: Scoping Review
Source: JMIR Mhealth Uhealth. 2026 Aug 10;14:e85197. doi: 10.2196/85197 (PMC13456309; doi:10.2196/85197)
Supplement: Supplementary file 3 [file mhealth-v14-e85197-s002.docx]

| Time Frame | 2013-2023 |
| --- | --- |
| Language | English |
| Type of Technology | Wearables (smartwatch, blood pressure monitor, etc.), Smart health device (weighing scale, etc.), PC and laptop computer, Smartphone, Smartphone applications (mood tracker, diet tracker, period tracker, etc.) excluding implants |
| Stakeholders | Patient and HCP |
| Publication Type | Peer-reviewed journal or conference article excluding meta-research (i.e., review papers) and study protocols |
| Initiation of Self-Tracking | Initiated by participants or patients or by clinicians |
| Focus of Study | PGHD integration for shared decision-making during in-person consultations or remote monitoring. |
