# Supplementary material for: Barriers and Enablers to Integrating Patient-Generated Health Data in Shared Decision-Making From Health Care Professional and Patient Perspectives: Scoping Review
Source: JMIR Mhealth Uhealth. 2026 Aug 10;14:e85197. doi: 10.2196/85197 (PMC13456309; doi:10.2196/85197)
Supplement: Supplementary file 4 [file mhealth-v14-e85197-s003.docx]

| Themes | Code | Explanation |
| --- | --- | --- |
| Design & Technology | Usability | A system which is easy to learn, easy to use and offers good user experience |
|  | Accessibility | Considering needs of a diverse population group, including excluded populations. |
|  | Technical Reliability | Robustness of technology which prevents it from errors or failures which could influence stability and reliability of device |
| Patient Provider Relationship | Trust | Patient's trust in HCPs and Vice Versa |
|  | Better communication | Improved communication between HCP and patients |
|  | Collaborative care | Interactions between patients or with doctors to improve health condition. |
|  | Better picture of patients | Allows HCPs to have a better view of a patient's condition with availability of additional data. |
|  | Well-informed patient | patients who are aware about their healthcare conditions prior to consultations |
|  | Expectations over PGHD Use | Agreement between HCP and Patient how PGHD is tracked and used during consultations |
|  | Shift of Responsibility | Increase in responsibility of doctors because of integrating and interpreting PGHD |
|  | Better Diagnosis | Allows HCPs to offer better diagnoses because of the increased information about the patient's condition |
|  | Accountability and Motivation of Patient | patients have a better sense of accountability because of having access to PGHD |
|  | Transparency of data driven decisions. | patients are interested in knowing how doctors made the decision using PGHD |
|  | Objectify information | PGHD helps to objectify subjective data provided by patients |
|  | Personalized Care | HCPs can offer tailored care based on the PGHD |
| Patient Perceptions and Characteristics | Health literacy | patients' ability to find, understand, and use information make health-related decisions. |
|  | Self-motivation | Patient's motivation to self-treat by monitoring themselves |
|  | Burden of tracking (workload) | Increased workload for patients from tracking |
|  | Negative repercussions (of tracking) | Anxiety, Depression and Obsession with Data |
|  | Altruism | Patient's willingness to share their PGHD to improve research and innovation in healthcare sector |
|  | Selective Disclosure | Patient's privacy concerns about their behavior or conditions which prevents them from revealing information about themselves. |
|  | Non-compliance | Patients are unwilling to use the technology because of increased transparency and accountability |
|  | Acceptance of technology | Patient's willingness to adopt technologies for tracking and technology acceptance. |
|  | Habit of tracking | Patient’s ability to remember to self-track actively |
|  | Introspectiveness | Patient's ability to engage with their PGHD to improve self-treatment of their conditions |
| Organizational | Resource | HCPs not having enough time to look through the data or have too much workload |
|  | Collaboration and Productivity | Allows for data-driven collaboration between multiple stakeholders: doctors, patients, and caregivers. |
|  | Process, Tools and Policies | HCPs share factors related to workplace policies |
|  | Prep for consultation | Allow HCPs to have access to the PGHD before the consultation to prepare beforehand. |
| Data | Health Data Literacy | HCPs and patients being equipped with the relevant knowledge and technical skillsets to make sense of the PGHD. |
|  | Security and Privacy | Patients' and HCP's concerns about safekeeping of data. |
|  | Context | Having information about the context in which the PGHD was collected. |
|  | Sensemaking | HCPs and patients being able to make sense of the PGHD. |
|  | Trustworthiness | Trust and reliability of the PGHD which was collected. |
|  | Data-driven workflow | Factors involving data actionability, data standardization, data integration into EMR/EHR and data access. |
|  | Relevance | Presenting only relevant PGHD data during consultations without overwhelming HCPs with unnecessary data. |
|  | Insufficient Data | Incomplete data for HCPs to make decisions. |
| Medical Ethics/Law | Patient Autonomy | Allowing patients to make decisions on their own without pressure from HCPs. |
|  | PGHD’s Value as evidence | Reliability of PGHD in clinical context for decision making. |
|  | Insurance | Use of PGHD for insurance sector. |
