# Supplementary material for: Barriers and Enablers to Integrating Patient-Generated Health Data in Shared Decision-Making From Health Care Professional and Patient Perspectives: Scoping Review
Source: JMIR Mhealth Uhealth. 2026 Aug 10;14:e85197. doi: 10.2196/85197 (PMC13456309; doi:10.2196/85197)
Supplement: Supplementary file 5 [file mhealth-v14-e85197-s004.docx]

### Characteristics of Included Papers

Note: The study IDs in the table below should be used in conjunction with the excel file containing the enablers and barriers.

| **Study ID** | **Reference** | **Paper Title** | **Publication Venue (Name of conference or journal)** | **Country of Origin** | **Sample Size** | **Medical Condition (Acute/Chronic)** | **PGHD Generating Technology(ies)** |
| --- | --- | --- | --- | --- | --- | --- | --- |
| **1** | Bardram et al 2023 [57] | DiaFocus: A Personal Health Technology for Adaptive Assessment in Long-Term Management of Type 2 Diabetes | ACM Transactions on Computing for Healthcare | Denmark | Patients (n=12) | T2D (Chronic) | Smartphone App, Diafocus Mobile sensing platform, Accu-Chek guide BGM. |
| **2** | West et al 2022 [48] | Personalized vascular healthcare: insights from a large international survey | European Heart Journal | USA, UK, China, Japan, France, Germany, India, Italy, and Brazil | Patients (n=961) HCPs (n=345) | Vascular Diseases (Chronic) | Not specified/N.A. |
| **3** | Wendrich and Krabbenborg 2022 [58] | Digital Self-monitoring of Multiple Sclerosis: Interview Study With Dutch Health Care Providers on the Expected New Configuration of Roles and Responsibilities | JMIR mhealth uhealth. | Netherlands | HCPs (n=28) | Multiple Sclerosis (Chronic) | Smartphone self-monitoring application - MS sherpa. |
| **4** | Seals et al 2022 [59] | ‘Are They Doing Better In The Clinic Or At Home?’: Understanding Clinicians’ Needs When Visualizing Wearable Sensor Data Used In Remote Gait Assessments For People With Multiple Sclerosis | CHI | USA | HCPs (n=10) | Multiple Sclerosis (Chronic) | Dashboard for Clinicians. |
| **5** | Oh et al 2022 [6] | Patients Waiting for Cues: Information Asymmetries and Challenges in Sharing Patient-Generated Data in the Clinic | CSCW | USA | Patients (n=57) | General | Not specified/N.A. |
| **6** | Jo et al 2022 [60] | GeniAuti: Toward Data-Driven Interventions to Challenging Behaviors of Autistic Children through Caregivers' Tracking | CSCW | South Korea | Parents and Children with Autism (n=38) HCP (n=3) Caregivers (n=6) | Autism (Chronic) | GeniAuti - a system that assists caregivers in systematically tracking their children’s challenging behaviors and their response strategies for data-driven discussion with clinical experts. |
| **7** | Aarts et al 2022 [61] | Snoozy: A Chatbot-Based Sleep Diary for Children Aged Eight to Twelve | IDC | Netherlands | Clinicians (n=6) Parents (n=6) Children (n=6) | Insomnia (Chronic) | Chatbot as a sleep diary. |
| **8** | White et al 2021[62] | Remote Measurement in Rheumatoid Arthritis: Qualitative Analysis of Patient Perspectives | JMIR | UK | Patients (n=9) | Rheumatoid Arthritis (Chronic) | Not specified/N.A. |
| **9** | Vaughn 2021 [45] | Seriously ill pediatric patient, parent, and clinician perspectives on visualizing symptom data | Journal of the American Medical Informatics Association | USA | Children (n=14) Parents (n=14) HCP (n=30) | Sickle Cell Disease, Cancer, and Bone Marrow Transplant (Chronic) | Hardware (Apple Watch) and Mobile App + Software (Visualization evaluation). |
| **10** | Richter et al 2021 [63] | Mobile App-based documentation of patient-reported outcomes — 3-months results  from a proof-of-concept study on modern rheumatology patient management | Journal on RA | Germany | Patients (n=111) | Rheumatoid Arthritis (Chronic) | Mobile Application. |
| **11** | Rainey et al 2021 [64] | Data Contribution Summaries for Patient Engagement in Multi-Device Health Monitoring Research | Ubicomp | United Kingdom and European Union | Patients (n=24) HCPs (n=9) | Huntington's disease, Parkinson's disease, inflammatory bowel disease (Chronic) | Mobile application with multiple sensing devices. |
| **12** | Kim et al 2021 [31] | Clinicians and Older Adults’ Perceptions of the Utility of Patient-Generated Health Data in Caring for Older Adults: Exploratory Mixed Methods Study | JMIR | Canada | Older Adults (n=5) HCPs (n=4) | General | Not specified/N.A. |
| **13** | Hoefer et al 2021 [65] | The Multiplicative Patient and the Clinical Workflow: Clinician Perspectives on Social Interfaces for Self-Tracking and Managing Bipolar Disorder | DIS | USA | HCPs (n=17) | Bipolar Disorder (Chronic) | Mobile Application. |
| **14** | Gupta et al 2020 [66] | Oh, I Didn't Do a Good Job: How Objective Data Affects Physiotherapist-Patient Conversations for Arthritis Patients | Pervasive Health | Canada | HCPs (n=7) | Arthritis (Chronic) | Web Application (FitViz). |
| **15** | Figueiredo et al 2021 [56] | Using Data to Approach the Unknown: Patients' and Healthcare Providers? Data Practices in Fertility Challenges | CSCW | USA | Patients (n=14) HCPs (n=5) | Infertility (Chronic) | Paper, websites, digital spreadsheets, fertility apps, wearables, or a combination of these. |
| **16** | Caldeira et al 2021 [44] | Towards Supporting Data-Driven Practices in Stroke Telerehabilitation Technology | CSCW | USA | Patients (n=10) HCPs (n=4) | Stroke (Chronic) | Game based Tele rehabilitation. |
| **17** | Zhu et al 2020 [67] | Prehabilitation: Care Challenges and Technological Opportunities | CHI | USA | HCPs (n=12) | Cancer, Facial nerve Palsy (Acute), Elderly, ENT, Knee Replacement (Acute and Chronic) | Varied Sensing and Medical Devices. |
| **18** | Wu et al 2020 [93] | Clinician Perspectives and Design Implications in Using Patient-Generated Health Data to Improve Mental Health Practices: Mixed Methods Study | JMIR | USA | HCPs (n=12) | Mental Health (Chronic) | Mobile Applications |
| **19** | Luo et al 2020 [68] | Interrelationships Between Patients’ Data Tracking Practices, Data Sharing Practices, and Health Literacy: Onsite Survey Study | JMIR | USA | Patients (n=109) | General | Not specified/N.A. |
| **20** | Lavallee et al 2020 [9] | mHealth and patient generated health data: stakeholder perspectives on opportunities and barriers for transforming healthcare | mHealth | USA | Healthcare Consumers (n=21) HCP (n=15) Healthcare Administrators (n=5) | General | Not specified/N.A. |
| **21** | Jongsma et al 2020 [69] | User Experiences with and Recommendations for Mobile Health Technology for Hypertensive Disorders of Pregnancy: Mixed Methods Study | JMIR mhealth uhealth | Netherlands | Patients (n=63) | Pregnancy (Acute) | -Automated blood pressure monitor with Bluetooth connection  -Smartphone app for iOS users and a web-based portal for Android users. |
| **22** | Bhat and Kumar 2020 [43] | Sociocultural Dimensions of Tracking Health and Taking Care | CSCW | India | Patients (n=18) HCPs (n=8) | Cardiovascular Disease (Chronic) | Smartphone App |
| **23** | Alpert et al 2020 [70] | Secondary care provider attitudes towards patient- generated health data from smartwatches | npj digital medicine | USA | HCPs (n=12) | General | Hardware and Software. |
| **24** | Tendedez et al 2019 [71] | The Issue with That Sort of Data?': Clinicians' Accountability Concerns Around COPD Self-Monitoring Tools | CSCW | UK | HCPs (n=4) | Chronic Obstructive Pulmonary Disease (Chronic) | Software. |
| **25** | Schroeder et al 2019 [47] | Examining Opportunities for Goal-Directed Self-Tracking to Support Chronic Condition Management | ACM IMWUT | USA | Patients (n= 14) HCPs (n=5) | Migraine (Chronic) | Software. |
| **26** | Ng et al 2019 [72] | Provider Perspectives on Integrating Sensor-Captured Patient-Generated Data in Mental Health Care | CSCW | USA | HCPs (n=17) | PTSD (Chronic) | Hardware - Fitbit |
| **27** | Iott et al 2019 [73] | Clinician Perspectives on the User Experience, Configuration, and Scope of Use of a Patient Reported Outcomes (PRO) Dashboard | PervasiveHealth | USA | HCPs (n=25) | Cancer (Chronic) | Software - PRO Data Dashboard tool |
| **28** | Hue et al 2019 [74] | Exploring the Future Role of Self-Tracking Data in the Rheumatology Clinic | Healthcare of the Future | UK | Patients (n=28) | Ankylosing spondylitis (Chronic) | Not specified/N.A. |
| **29** | Chung et al 2019 [34] | Identifying and Planning for Individualized Change: Patient-Provider Collaboration Using Lightweight Food Diaries in Healthy Eating and Irritable Bowel Syndrome | ACM IMWUT | USA | Healthy participants (n=17) Patients (n=16) HCP(n=15) | Irritable Bowel Syndrome (Chronic) | Software –  (1) a mobile app supporting in -the-moment, low-burden food capture  (2) a web app presenting relationships between food and health goals,  (3) a pre-visit note asking for participant summary about their data and their goals and expectations for the visit. |
| **30** | Abdolkhani et al 2019 [13] | Patient-generated health data management and quality challenges in remote patient monitoring | JAMIA Open | Australia | HCPs (n=9) Health information professionals (n=4) Remote patient monitoring solution providers (n=7) | Diabetes, Cardiac Arrhythmia, Sleep Disorder (Chronic) | Diabetes: Continuous glucose monitoring (CGM) devices and insulin pumps; consumer wearables were various types of fitness trackers Cardiac arrhythmia: an event monitoring wearable sleep disorder: the medical wearable was a sleep monitoring/ activity watch. |
| **31** | Trauth et al 2018 [75] | Technologized Talk: Wearable Technologies, Patient Agency, and Medical Communication in Healthcare Settings | International Journal of Sociotechnology and Knowledge Development | USA | Patients (n=84) | General Health management (Chronic and Acute) | Not specified. |
| **32** | West et al 2018 [12] | Common Barriers to the Use of Patient-Generated Data Across Clinical Settings | CHI | UK | HCPs (n=13) | General Health management (Chronic and Acute) | Not specified. |
| **33** | Tendedez et al 2018 [76] | Scoping the Design Space for Data Supported Decision Making Tools in Respiratory Care: Needs, Barriers and Future Aspirations | PervasiveHealth | UK | HCPs (n=4) Health information professionals (n=6) | Chronic Obstructive Pulmonary Disease (Chronic) | Not specified. |
| **34** | Reading et al 2018 [77] | Factors influencing sustained engagement with ECG self-monitoring: perspectives from Patients and health care providers | Journal of Applied clinical informatics | USA | Patients (n=13) Research coordinators (n=2) | Atrial fibrillation (chronic) | Mobile electrocardiogram (ECG) monitor and smartphone application. |
| **35** | Piras and Miele 2017 [46] | Clinical self-tracking and monitoring technologies: negotiations in the ICT mediated patient–provider relationship | HEALTH SOCIOLOGY REVIEW journal | Italy | Parents (n=8) Children/Adolescents (n=4) Patients (Adults) (n=17) HCPs (n=3) Health information professionals (n=3) | T1D (Chronic) | Mobile application and Support Platform. |
| **36** | Ng et al 2018 [78] | Veterans’ Perspectives on Fitbit Use in Treatment for Post-Traumatic Stress Disorder: An Interview Study | JMIR MENTAL HEALTH | USA | Patients (n=13) | Mental Health (Chronic) PTSD | Fitbit software and Charge HR. |
| **37** | Mishra et al 2018 [79] | Supporting Collaborative Health Tracking in the Hospital: Patients’ Perspectives | CHI | USA | Patients and caregivers (n=30) | General | PGHD tracking platform |
| **38** | Gabriels and Moerenhout 2018 [80] | Exploring entertainment medicine and professionalization of self-care: interview study among doctors on the potential effects of digital self-tracking | JMIR | Belgium | HCPs (n=12) | General | N.A. |
| **39** | Zhu et al 2017 [81] | Making Space for the Quality Care: Opportunities for Technology in Cognitive Behavioral Therapy for Insomnia | CHI | USA | Patients (n=17) HCPs (n=11) | Insomnia (Chronic) | N.A. |
| **40** | Schroeder et al 2017 [82] | Supporting Patient-Provider Collaboration to Identify Individual Triggers Using Food and Symptom Journals | CSCW | USA | Patients (n=10) HCPs (n=10) | IBS(Chronic) | N.A. |
| **41** | Raj et al 2017 [83] | Understanding Individual and Collaborative Problem-Solving with Patient-Generated Data: Challenges and Opportunities | CSCW | USA | Interviews = Patients and Caregivers (n=5), Caregiver (n=1), Patients (n=8), Clinicians (n=2) Focus group = Clinicians (n=4) Observations = Clinic sessions (n=5), Phone call sessions (n=6) | Diabetes (Chronic) | Continuous Glucose Monitor, Insulin Pump |
| **42** | Malu and Findlater 2017 [84] | Sharing automatically tracked activity data: implications for therapists and people with mobility impairments | PervasiveHealth | USA | Patients (n=10) HCPs (n=10) | Mobility Impairment (Chronic) | Software and Hardware. |
| **43** | Kim et al 2017 [85] | Prescribing 10,000 Steps Like Aspirin: Designing a Novel Interface for Data-Driven Medical Consultations | CHI | South Korea | HCPs (n=4) Health information professionals (n=5) Others (n=11) | General Health (Acute and Chronic) | Not specified/N.A. |
| **44** | Kelley et al 2017 [86] | Self-Tracking for Mental Wellness: Understanding Expert Perspectives and Student Experiences | CHI | USA | Student Health Professionals (n=14) Students (n=297) | Mental Health (Long Term) | Not specified/N.A. |
| **45** | Zhu et al 2016 [7] | Sharing Patient-Generated Data in Clinical Practices: An Interview Study | AMIA | USA | Patients (n=12) HCPs (n=9) | General | Not specified/N.A. |
| **46** | West et al 2016 [87] | The Quantified Patient in the Doctor's Office: Challenges & Opportunities | CHI | USA | HCPs (n=10) | General | Excel Sheet iPhone App |
| **47** | Kim et al 2016 [88] | "My Doctor is Keeping an Eye on Me!": Exploring the Clinical Applicability of a Mobile Food Logger | CHI | Korea | Patients (n=20) HCPs (n=11) | Lifestyle Diseases (Chronic) | Software - mfood logging application. |
| **48** | Cohen et al 2016 [89] | Integrating Patient-Generated Health Data Into Clinical Care Settings or Clinical Decision-Making: Lessons Learned From Project HealthDesign | JMIR | USA | Study Team members (n=13) HCPs (n=12) | Diverse Health issues: Cognitive Decline, Moderate to severe asthma, adolescent behavioral health, Crohn disease, health issues with premature infants | Software. |
| **49** | Chung et al 2016 [21] | Boundary Negotiating Artifacts in Personal Informatics: Patient-Provider Collaboration with Patient-Generated Data | CSCW | USA | Patients (n=229) HCPs (n=21) | IBS, Obesity (Chronic) | Not specified/N.A. |
| **50** | Ryokai et al 2015 [90] | Communicating and interpreting wearable sensor data with health coaches | PervasiveHealth | USA | Participants (n=5) HCPs (n=4) | Health Coaching | Hardware and Software. |
| **51** | Chung et al 2015 [30] | More Than Telemonitoring: Health Provider Use and Nonuse of Life-Log Data in Irritable Bowel Syndrome and Weight Management | JMIR | USA | HCP (n=21) | IBS, Weight Management (Chronic) | Software Dashboard (Fitbit, Gut Guru, Health Report (Conceptual app to allow Patients track symptoms between visits). |
| **52** | Ancker et al 2015 [91] | “You Get Reminded You’re a Sick Person”: Personal Data Tracking and Patients with Multiple Chronic Conditions | JMIR | USA | Patients (n=22) HCPs (n=7) | MCC (including diabetes, HIV, heart disease, depression, and many others) | Hardware - Blood Glucose and pressure monitor  Software - medication, weight, food. |
| **53** | Nundy et al 2014 [92] | Using Patient-Generated Health Data from Mobile Technologies for Diabetes Self-Management Support | Journal of Diabetes Science and Technology | USA | HCPs (n=12) | Diabetes (Chronic) | Software (SMS and CareSmarts Dashboard |

**References**

57. Bardram JE, Cramer-Petersen C, Maxhuni A, et al. DiaFocus: a personal health technology for adaptive assessment in long-term management of type 2 diabetes. ACM Trans Comput Healthcare. Apr 30, 2023;4(2):1-43. [doi: 10.1145/ 3586579]

48. West NEJ, Juneja M, Pinilla N, et al. Personalized vascular healthcare: insights from a large international survey. Eur Heart J Suppl. Nov 2022;24(Suppl H):H8-H17. [doi: 10.1093/eurheartjsupp/suac052] [Medline: 36382003]

58. Wendrich K, Krabbenborg L. Digital self-monitoring of multiple sclerosis: interview study with dutch health care providers on the expected new configuration of roles and responsibilities. JMIR mHealth uHealth. 2022;10(4):e30224. [doi: 10.2196/30224]

59. Seals A, Pilloni G, Kim J, et al. ‘Are they doing better in the clinic or at home?’: Understanding clinicians’ needs when visualizing wearable sensor data used in remote gait assessments for people with multiple sclerosis. Presented at: CHI ’22: Proceedings of the 2022 CHI Conference on Human Factors in Computing Systems; Apr 30 to May 6, 2022:1-16; New Orleans, LA. [doi: 10.1145/3491102.3501989]

6. Oh CY, Luo Y, Jean B, Choe EK. Patients waiting for cues: information asymmetries and challenges in sharing patientgenerated data in the clinic. Proc ACM Hum-Comput Interact. 2022;6(CSCW1):1-23. [doi: 10.1145/3512954]

60. Jo E, Park S, Bang H, et al. GeniAuti: toward data-driven interventions to challenging behaviors of autistic children through caregivers’ tracking. Proc ACM Hum-Comput Interact. Mar 30, 2022;6(CSCW1):1-27. [doi: 10.1145/3512939]

61. Aarts T, Markopoulos P, Giling L, Vacaretu T, Pillen S. Snoozy: a chatbot-based sleep diary for children aged eight to twelve. Presented at: IDC ’22: Proceedings of the 21st Annual ACM Interaction Design and Children Conference; Jun 27 to Jul 30, 2022; Braga, Portugal. [doi: 10.1145/3501712.3529718]

62. White KM, Ivan A, Williams R, Galloway JB, Norton S, Matcham F. Remote measurement in rheumatoid arthritis: qualitative analysis of patient perspectives. JMIR Form Res. Mar 9, 2021;5(3):e22473. [doi: 10.2196/22473] [Medline: 33687333]

45. Vaughn J, Kamkhoad D, Shaw RJ, Docherty SL, Subramaniam AP, Shah N. Seriously ill pediatric patient, parent, and clinician perspectives on visualizing symptom data. J Am Med Inf Assoc. Jul 14, 2021;28(7):1518-1525. [doi: 10.1093/ jamia/ocab037] [Medline: 33712836]

63. Richter JG, Nannen C, Chehab G, et al. Mobile app-based documentation of patient-reported outcomes — 3-months results from a proof-of-concept study on modern rheumatology patient management. Arthritis Res Ther. Apr 19, 2021;23(1):121. [doi: 10.1186/s13075-021-02500-3] [Medline: 33874994]

64. Rainey J, Verweij D, Dodds C, et al. Data contribution summaries for patient engagement in multi-device health monitoring research. Presented at: UbiComp/ISWC ’21 Adjunct: Adjunct Proceedings of the 2021 ACM International Joint Conference on Pervasive and Ubiquitous Computing and Proceedings of the 2021 ACM International Symposium on Wearable Computers; Sep 21-26, 2021. [doi: 10.1145/3460418.3479371]

31. Kim B, Ghasemi P, Stolee P, Lee J. Clinicians and older adults’ perceptions of the utility of patient-generated health data in caring for older adults: exploratory mixed methods study. JMIR Aging. Nov 5, 2021;4(4):e29788. [doi: 10.2196/ 29788]

65. Hoefer MJD, Van Kleunen L, Goodby C, Blackburn LB, Panati P, Voida S. The multiplicative patient and the clinical workflow: clinician perspectives on social interfaces for self-tracking and managing bipolar disorder. Presented at: DIS ’21: Proceedings of the 2021 ACM Designing Interactive Systems Conference; Jun 28 to Jul 2, 2021. [doi: 10.1145/ 3461778.3461995]

66. Gupta A, Heng T, Shaw C, Gromala D, Leese J, Li L. Oh, I didn’t do a good job: how objective data affects physiotherapist-patient conversations for arthritis patients. Presented at: PervasiveHealth ’20: Proceedings of the 14th EAI International Conference on Pervasive Computing Technologies for Healthcare; May 18-20, 2020; Atlanta, GA. [doi: 10.1145/3421937.3421991]

56. Figueiredo MC, Su HI, Chen Y. Using data to approach the unknown: patients' and healthcare providers? Data practices in fertility challenges. Proc ACM Hum-Comput Interact. Jan 5, 2021;4(CSCW3):1-35. [doi: 10.1145/3432926]

44. Caldeira C, Figueiredo MC, Dodakian L, de Souza CRB, Cramer SC, Chen Y. Towards supporting data-driven practices in stroke telerehabilitation technology. Proc ACM Hum-Comput Interact. Apr 13, 2021;5(CSCW1):1-33. [doi: 10.1145/ 3449099]

67. Zhu H, Moffa ZJ, Gui X, Carroll JM. Prehabilitation: care challenges and technological opportunities. Presented at: CHI ’20: Proceedings of the 2020 CHI Conference on Human Factors in Computing Systems; Apr 25-30, 2020; Honolulu, HI. [doi: 10.1145/3313831.3376594]

93. Wu DTY, Xin C, Bindhu S, et al. Clinician perspectives and design implications in using patient-generated health data to improve mental health practices: mixed methods study. JMIR Form Res. Aug 7, 2020;4(8):e18123. [doi: 10.2196/ 18123] [Medline: 32763884]

68. Luo Y, Oh CY, Jean BS, Choe EK. Interrelationships between patients’ data tracking practices, data sharing practices, and health literacy: onsite survey study. J Med Internet Res. Dec 22, 2020;22(12):e18937. [doi: 10.2196/18937] [Medline: 33350960]

9. Lavallee DC, Lee JR, Austin E, et al. mHealth and patient generated health data: stakeholder perspectives on opportunities and barriers for transforming healthcare. mHealth. 2020;6:8. [doi: 10.21037/mhealth.2019.09.17] [Medline: 32190619]

69. Jongsma KR, van den Heuvel JFM, Rake J, Bredenoord AL, Bekker MN. User experiences with and recommendations for mobile health technology for hypertensive disorders of pregnancy: mixed methods study. JMIR mHealth uHealth. Aug 4, 2020;8(8):e17271. [doi: 10.2196/17271] [Medline: 32749225]

43. Bhat KS, Kumar N. Sociocultural dimensions of tracking health and taking care. Proc ACM Hum-Comput Interact. Oct 14, 2020;4(CSCW2):1-24. [doi: 10.1145/3415200]

70. Alpert JM, Manini T, Roberts M, et al. Secondary care provider attitudes towards patient generated health data from smartwatches. NPJ Digit Med. 2020;3(1):27. [doi: 10.1038/s41746-020-0236-4] [Medline: 32140569]

71. Tendedez H, Ferrario MA, McNaney R. The issue with that sort of data?”: Clinicians’ accountability concerns around COPD self-monitoring tools. Presented at: 2019 Computer Supported Cooperative Work and Social Computing Companion Publication (CSCW ’19 Companion Proceedings); Nov 9-13, 2019; Austin, TX. [doi: 10.1145/3311957. 3359464]

47. Schroeder J, Karkar R, Murinova N, Fogarty J, Munson SA, Munson SA. Examining opportunities for goal-directed self-tracking to support chronic condition management. Proc ACM Interact Mobile Wearable Ubiquitous Technol. Dec 2019;3(4):1-26. [doi: 10.1145/3369809] [Medline: 32656490]

72. Ng A, Kornfield R, Schueller SM, Zalta AK, Brennan M, Reddy M. Provider perspectives on integrating sensorcaptured patient-generated data in mental health care. Proc ACM Hum-Comput Interact. Nov 7, 2019;3(CSCW):1-25. [doi: 10.1145/3359217]

73. Iott B, Caverly T, Fishstrom A, King D, Meng G, Flynn A. Clinician perspectives on the user experience, configuration, and scope of use of a patient reported outcomes (PRO) dashboard. Presented at: PervasiveHealth’19: Proceedings of the 13th EAI International Conference on Pervasive Computing Technologies for Healthcare; May 20-23, 2019:21-30; Trento, Italy. [doi: 10.1145/3329189.3329198]

74. Hue W, Jones S, Sengupta R. Exploring the future role of self-tracking data in the rheumatology clinic. Stud Health Technol Inf. 2019;259(33–38):33-38. [Medline: 30923269]

34. Chung CF, Wang Q, Schroeder J, et al. Identifying and planning for individualized change: patient-provider collaboration using lightweight food diaries in healthy eating and irritable bowel syndrome. Proc ACM Interact Mobile Wearable Ubiquitous Technol. Mar 2019;3(1):7. [doi: 10.1145/3314394] [Medline: 31080941]

13. Abdolkhani R, Gray K, Borda A, DeSouza R, DeSouza R. Patient-generated health data management and quality challenges in remote patient monitoring. JAMIA Open. Dec 2019;2(4):471-478. [doi: 10.1093/jamiaopen/ooz036] [Medline: 32025644]

75. Trauth E, Browning ER, Browning ER. Technologized talk: wearable technologies, patient agency, and medical communication in healthcare settings. Int J Sociotechnol Knowl Dev. Jul 1, 2018;10(3):1-26. [doi: 10.4018/IJSKD. 2018070101]

12. West P, Van Kleek M, Giordano R, Weal MJ, Shadbolt N. Common barriers to the use of patient-generated data across clinical settings. Presented at: CHI ’18: Proceedings of the 2018 CHI Conference on Human Factors in Computing Systems; Apr 21-26, 2018:1-13; Montreal, Quebec, Canada. [doi: 10.1145/3173574.3174058]

76. Tendedez H, McNaney R, Ferrario MA, Whittle J. Scoping the design space for data supported decision-making tools in respiratory care. Presented at: Proceedings of the 12th EAI International Conference on Pervasive Computing Technologies for Healthcare; May 21-24, 2018:217-226; New York, NY. [doi: 10.1145/3240925.3240983]

77. Reading M, Baik D, Beauchemin M, Hickey KT, Merrill JA. Factors influencing sustained engagement with ECG selfmonitoring: perspectives from patients and health care providers. Appl Clin Inf. Oct 2018;9(4):772-781. [doi: 10.1055/s0038-1672138] [Medline: 30304745]

46. Piras EM, Miele F. Clinical self-tracking and monitoring technologies: negotiations in the ICT-mediated patient– provider relationship. Health Sociol Rev. Jan 2, 2017;26(1):38-53. [doi: 10.1080/14461242.2016.1212316]

78. Ng A, Reddy MC, Zalta AK, Schueller SM. Veterans’ perspectives on Fitbit use in treatment for post-traumatic stress disorder: an interview study. JMIR Ment Health. Jun 15, 2018;5(2):e10415. [doi: 10.2196/10415] [Medline: 29907556]

79. Mishra SR, Miller AD, Haldar S, et al. Supporting collaborative health tracking in the hospital. Presented at: CHI ’18: Proceedings of the 2018 CHI Conference on Human Factors in Computing Systems; Apr 21-26, 2018; Montreal, Quebec, Canada. [doi: 10.1145/3173574.3174224]

80. Gabriels K, Moerenhout T. Exploring entertainment medicine and professionalization of self-care: interview study among doctors on the potential effects of digital self-tracking. J Med Internet Res. Jan 12, 2018;20(1):e10. [doi: 10. 2196/jmir.8040] [Medline: 29330140]

81. Zhu H, Luo Y, Luo Y, Choe EK. Making space for the quality care: opportunities for technology in cognitive behavioral therapy for insomnia. Presented at: CHI ’17: Proceedings of the 2017 CHI Conference on Human Factors in Computing Systems; May 6-11, 2017:5773-5786; [doi: 10.1145/3025453.3025549]

82. Schroeder J, Hoffswell J, Chung CF, Fogarty J, Munson S, Zia J. Supporting patient-provider collaboration to identify individual triggers using food and symptom journals. Presented at: CSCW ’17: Proceedings of the 2017 ACM Conference on Computer Supported Cooperative Work and Social Computing; Feb 25 to Mar 1, 2017:1726-1739; Portland, OR. [doi: 10.1145/2998181.2998276]

83. Raj S, Newman MW, Lee JM, Ackerman MS. Understanding individual and collaborative problem-solving with patientgenerated data. Proc ACM Hum-Comput Interact. Dec 6, 2017;1(CSCW):1-18. [doi: 10.1145/3134723]

84. Malu M, Findlater L. Sharing automatically tracked activity data: implications for therapists and people with mobility impairments. Presented at: PervasiveHealth ’17: Proceedings of the 11th EAI International Conference on Pervasive Computing Technologies for Healthcare; May 23-26, 2017:136-145; Barcelona, Spain. [doi: 10.1145/3154862.3154864]

85. Kim Y, Heo E, Lee H, et al. Prescribing 10,000 steps like aspirin: designing a novel interface for data-driven medical consultations. Presented at: CHI ’17: Proceedings of the 2017 CHI Conference on Human Factors in Computing Systems; May 6-11, 2017:5787-5799; Denver, CO. [doi: 10.1145/3025453.3025570]

86. Kelley C, Lee B, Wilcox L. Self-tracking for mental wellness: understanding expert perspectives and student experiences. Presented at: CHI ’17: Proceedings of the 2017 CHI Conference on Human Factors in Computing Systems; May 6-11, 2017; Denver, CO. [doi: 10.1145/3025453.3025750]

7. Zhu H, Colgan J, Reddy M, Choe EK. Sharing patient-generated data in clinical practices: an interview study. AMIA Annu Symp Proc. 2016;2016:1303-1312. [Medline: 28269928]

87. West P, Giordano R, Van Kleek M, Shadbolt N. The quantified patient in the doctor’s office: challenges & opportunities. Presented at: CHI ’16: Proceedings of the 2016 CHI Conference on Human Factors in Computing Systems; May 7-12, 2016; San Jose, CA. [doi: 10.1145/2858036.2858445]

88. Kim Y, Ji S, Lee HJ, et al. “My doctor is keeping an eye on me!”: exploring the clinical applicability of a mobile food logger. Presented at: CHI ’16: Proceedings of the 2016 CHI Conference on Human Factors in Computing Systems; May 7-12, 2016:5620-5631; San Jose, CA. [doi: 10.1145/2858036.2858145]

89. Cohen DJ, Keller SR, Hayes GR, Dorr DA, Ash JS, Sittig DF. Integrating patient-generated health data into clinical care settings or clinical decision-making: lessons learned from Project HealthDesign. JMIR Hum Factors. Oct 19, 2016;3(2):e26. [doi: 10.2196/humanfactors.5919] [Medline: 27760726]

21. Chung CF, Dew K, Cole A, et al. Boundary negotiating artifacts in personal informatics: patient-provider collaboration with patient-generated data. Presented at: 9th ACM Conference on Computer-Supported Cooperative Work (CSCW 2002); Feb 27 to Mar 2, 2016:770-786; San Francisco, CA. [doi: 10.1145/2818048.2819926]

90. Ryokai K, Michahelles F, Kritzler M, Syed S. Communicating and interpreting wearable sensor data with health coaches. Presented at: 9th International Conference on Pervasive Computing Technologies for Healthcare; May 20-23, 2015:221-224; Istanbul, Turkey. [doi: 10.4108/icst.pervasivehealth.2015.259055]

30. Chung CF, Cook J, Bales E, Zia J, Munson SA. More than telemonitoring: health provider use and nonuse of life-log data in irritable bowel syndrome and weight management. J Med Internet Res. Aug 21, 2015;17(8):e203. [doi: 10.2196/ jmir.4364] [Medline: 26297627]

91. Ancker JS, Witteman HO, Hafeez B, Provencher T, Van de Graaf M, Wei E. “You get reminded you’re a sick person”: personal data tracking and patients with multiple chronic conditions. J Med Internet Res. Aug 19, 2015;17(8):e202. [doi: 10.2196/jmir.4209]

92. Nundy S, Lu CYE, Hogan P, Mishra A, Peek ME. Using patient-generated health data from mobile technologies for diabetes self-management support: provider perspectives from an academic medical center. J Diabetes Sci Technol. Jan 2014;8(1):74-82. [doi: 10.1177/1932296813511727] [Medline: 24876541]
